# Environmental Contamination and Mining Impact: Physico-Chemical and Biological Characterization of Propolis as an Indicator of Pollution in the Roșia Montană Area, Romania

**DOI:** 10.3390/plants14060866

**Published:** 2025-03-10

**Authors:** Mirel Glevitzky, Roxana Bostan, Mihaela Laura Vică, Gabriela-Alina Dumitrel, Mihai-Teopent Corcheş, Maria Popa, Ioana Glevitzky, Horea-Vladi Matei

**Affiliations:** 1 Faculty of Informatics and Engineering, “1 Decembrie 1918” University of Alba Iulia, 15-17 Unirii Street, 510009 Alba Iulia, Romania; mirel_glevitzky@yahoo.com (M.G.); roxana.bostan@uab.ro (R.B.); mihai.corches@uab.ro (M.-T.C.); mpopa@uab.ro (M.P.); 2 Sanitary Veterinary and Food Safety Directorate of Alba County, 7A Lalelor Street, 510217 Alba Iulia, Romania; 3 Department of Cellular and Molecular Biology, “Iuliu Hațieganu” University of Medicine and Pharmacy, 6 Louis Pasteur Street, 400012 Cluj-Napoca, Romania; hmatei@umfcluj.ro; 4 Institute of Legal Medicine, 400006 Cluj-Napoca, Romania; 5 Faculty of Industrial Chemistry and Environmental Engineering, Politehnica University Timişoara, 2 Victoriei Square, 300006 Timişoara, Romania; alina.dumitrel@upt.ro

**Keywords:** metal cations, soil, water, vegetables, contamination, propolis, total phenolics, total flavonoids, DPPH

## Abstract

Contamination with heavy metal ions from mining activities presents a major environmental issue. This study investigates pollution caused by heavy metals from mining, with a particular emphasis on toxic ions and essential ions for living organisms. It starts by analyzing the sources of pollution and its effects on soil, vegetation, water, and wildlife (propolis produced by honey bees living in natural environments). Propolis is an indicator of environmental contamination by metals, a natural and valuable product of natural ecosystems. As part of the investigation, the contamination with metal cations (Pb^2+^, Cu^2+^, Cd^2+^, Zn^2+^, As^3+^, Fe^2+^, and Sr^2+^) of the soil, cultivated vegetables (carrot, turnip, onion, potato) was monitored in 9 points in the Roșia Montană area, Romania, as well as the river that runs through the area. The maximum values of the parameters investigated were recorded in soil (108.32 mg/kg Pb^2+^, 23.06 mg/kg Cd^2+^, 102.17 mg/kg As^3+^), river water (11.00 µg/L Pb^2+^, 903.47 µg/L Cu^2+^, 60.13 µg/L Cd^2+^, 1903.08 µg/L Zn^2+^, 148.07 µg/L As^3+^, 44,024.08 µg/L Fe^2+^), vegetables (0.72 mg/kg Pb^2+^, 0.17 mg/kg Cd^2+^) and it was followed whether the same heavy metals are found in propolis (maximum values 10.14 mg/kg Pb^2+^, 6.32 mg/kg Cu^2+^, 0.158 mg/kg Cd^2+^, 6.0 Zn^2+^, 1.04 mg/kg As^3+^, 12.06 mg/kg Sr^2+^). The parameters analyzed for the river waters were pH, sulfates, the oxygen and nutrient regime, and microbial load. Additional investigations were carried out into the quality of these propolis samples: water activity, moisture, hygroscopicity, water solubility, volatile oils, oxidation index, measuring point, density, dry matter, material insoluble in ethanol, extractable with ethanol, ash, and wax. The highest values were 189.4 mg GAE/g for phenols, 84.31 mg QE/g for flavonoids, and 0.086 µg/mL for IC_50_ antioxidant activity. This study indicates that bee products, such as propolis, can be an indicator of pollution in mining areas.

## 1. Introduction

Metals and metalloids are naturally present in the environment but at low concentrations. Human activities, such as mining, have led to increased levels, exposing flora and fauna to dangerous concentrations [1]. Industrial, agricultural, and mining activities have led to an increase in environmental pollution [2]. Heavy metals are widely studied pollutants that continuously accumulate in the environment because of their stability and resistance to degradation [3].

Mining activities can significantly affect the environment, both during exploitation and long after the mines have closed. The main environmental problems arise from the waste generated during the extraction and processing of metals, which are major sources of soil, water, and air pollution [4]. In mining areas, these resources may contain heavy metals or other pollutants, and the analysis of bees and their products (such as honey, wax, or pollen) can reveal the level of contamination [5].

In mining areas, heavy metals such as lead (Pb), cadmium (Cd), mercury (Hg), and arsenic (As) accumulate in the soil, water, and air. The metals are detected both in the bodies of bees and in their products [6]. Monitoring bee products provides insights into the quality of the environment they originate from [7].

Bees are considered bioindicators of pollution [8,9,10,11,12,13,14,15] in mining areas because they collect pollen, nectar, and water from the surrounding environment, thus reflecting the health of the local ecosystem [16]. Bees exposed to pollution may show increased mortality, changes in behavior and health, and flight activity [17]. Bees influence the overall health of the ecosystem through pollination. If bees are affected by pollution, the entire ecological chain can be destabilized [18].

Propolis or “bee glue” is a resinous natural substance produced by bees (*Apis mellifera*) [19]. In the process of propolis preparation, the bees collect the resin from the leaves, buds, and bark [20]. This is mixed with salivary secretions and wax. Propolis is used to fill and seal cracks in the hive, providing protection against pathogens [21]. From a chemical point of view, propolis is composed of oil, wax, resin, pollen, amino acids, minerals, sugars, vitamins (B, C, E), flavonoids, phenols, aromatic compounds etc [22].

The name of the product comes from the Greek language (“pro” comes from “at the entrance to” and “polis” for “community/city”), being inspired by the fact that bees use this protective substance in the construction of the hive [23]. It is used as a natural remedy in multiple pathologies [24,25], especially in the treatment of absences, wounds; infections [26]. Propolis is a substance that is frequently used in apitherapy. It is an adjuvant in medical conditions such as gastrointestinal, allergic, viral, bacterial, dermatological, gynecological, infectious, oncological, and oral disorders [27].

The study aims to investigate the transfer of potentially toxic trace elements (Cu^2+^, Fe^2+^, Pb^2+^, Zn^2+^, Sr^2+^, As^3+^, and Cd^2+^) from the soil to plants grown under the impact of the Roșia Montană mining area by determining the contamination levels and assessing the dietary exposure of local residents. At the same time, the research also focuses on the role of bees, as vectors and practical and efficient tools for monitoring the effects of pollution on ecosystems, especially in these areas exposed to mining activities.

## 2. Results

### 2.1. Soil Analysis

The results obtained from laboratory analyses of soil samples collected from the 9 points in the Roșia Montană area are presented in Table 1.

To determine the content of metal cations in vegetation and the degree of translocation of them from the soil to the plants, soil samples were analyzed. From Table 1, for most metals, the concentration of normal values is exceeded in the soil of the Roșia Montană area. The maximum lead content recorded was 108.32 mg/kg in soil sample no. 8 taken in the NE of Roșia Montană. This value slightly exceeds the value established for the intervention threshold, which is 100 mg/kg. Zinc was present at a maximum concentration of 36.1 mg/kg not exceeding the maximum allowable concentration, and copper was within normal limits, the determined value being 23.0 mg/kg. The range of values for the Cd^2+^ content in the soil varies between 2.6 and 3.7 mg/kg, values that do not exceed the intervention threshold but are above the normal values of 1 mg/kg. Regarding the As^3+^ content, the maximum allowable limits were exceeded at all sampling points, the highest value being recorded in the northern area of Roșia Montană—102.1 mg/kg of As^3+^.

### 2.2. River Water

The water quality of the Roșia Montană River that flows through the area was investigated. The study was conducted by testing river water for inorganic contaminants, as well as common physico-chemical and microbiological parameters. The results obtained are presented in Table 2 and Table 3. The samples were taken at different times of the year, depending on the season, but also from one year to another. Thus, an attempt was made to capture the variations in the flow of the watercourse. In periods of high flow, following snowmelt, or in periods of abundant flow, the concentration of pollutants is usually lower.

These dissolved parameters (Cu^2+^, Fe^2+^, Zn^2+^, Pb^2+^, Cd^2+^, and As^3+^) are used in water quality monitoring, to assess the level of pollution, and to ensure compliance with environmental and health standards. If the Roșia Montană River has at the source (sample 1A) only exceedances of iron, zinc, and cadmium content, downstream the values indicate massive water pollution with copper, iron, zinc, cadmium, and arsenic regardless of the season.

The concentration of Cd^2+^ varied, ranging from 27.08 µg/L in February to 55.72 µg/L in August, with a notable increase in the warmer months. Zn^2+^ levels fluctuate between 48.42 µg/L in February and 65.18 µg/L in May, before slightly decreasing to 51.04 µg/L in August. Fe^2+^ shows relatively high concentrations, reaching 230.20 µg/L in August, and remains high throughout the year, with values ranging from 150.44 µg/L in February to 167.05 µg/L in November.

For River 1B, located in the middle of the river, the concentrations of Pb^2+^ become detectable starting in February, with values around 11.00 µg/L, and continue at similar levels throughout the year. The concentrations of Cu^2+^ were much higher compared to the 1A river, peaking at 903.47 µg/L in August, while May saw values of 820.23 µg/L. Cd^2+^ levels are also higher in River 1B, ranging from 50.89 µg/L in February to 60.13 µg/L in August, with the lowest value recorded in May at 20.51 µg/L. Zn^2+^ concentrations are significantly elevated in River 1B, especially in May and August, where values reach 1632.40 µg/L and 1890.27 µg/L, respectively. The levels of As^3+^ show a steady increase, from 117.20 µg/L in February to 148.07 µg/L in August. Concentrations of Fe^2+^ were alarmingly high, especially in May and August, with a peak of 44,024.08 µg/L in August.

When comparing these results to water quality classifications, which range from Class I (the highest water quality) to Class V (the lowest), the data from River 1A generally shows good water quality. The concentrations of most metals fall well below the limits for Class V, indicating relatively low pollution levels. However, the cadmium levels in River 1A during the summer months and the high iron concentrations remain a concern, with values falling under Class III in some cases.

In contrast, River 1B shows a significant decline in water quality, as the concentrations of Pb^2+^, Cu^2+^, Cd^2+^, Zn^2+^, As^3+^, and Fe^2+^ all exceed the limits for Class III and approach or surpass the thresholds for Class IV and Class V in some months, especially in August. This suggests that as the water travels downstream, it becomes more polluted, particularly with metals like copper, zinc, and iron.

Regarding the parameters: pH, sulfates, dissolved oxygen, biochemical oxygen demand, and chemical oxygen demand, at the source (sample 1A) the values obtained are normal, but downstream (sample 1B) the results indicate major water pollution.

The pH level is one of the most important indicators of water quality, reflecting its acidity or alkalinity. In River 1A, the pH varies from 6.34 in August to 7.80 in February, all within the acceptable range for most aquatic organisms. However, in River 1B, due to the acidic mine waters, the pH varies between 2.8 and 3.87, indicating extreme acidity, which can be harmful to aquatic life, disrupting the metabolic processes of fish and vegetables.

The concentrations of SO_4_^2−^ in the 1A river range from 21.01 mg/L in May to 48.22 mg/L in February. These levels are well within the limits of Class II water quality. However, in the Roșia Montană River water (downstream) varied between 1076.29 mg/L and 1307.1 mg/L, and the areas with high values were in the discharge area of the water from the mine drainage. This dramatic increase suggests contamination and can lead to water acidification and the formation of toxic sulfur compounds, which disrupt the aquatic ecosystem.

The determination of the indicators of the oxygen regime of river water does not indicate large variations. The water of the Roșia Montană River, which is in a mountainous area, undergoes a natural aeration process, during which it is enriched with oxygen because of turbulence and intense contact with the atmosphere.

Dissolved oxygen is essential for supporting life in aquatic ecosystems, with higher values indicating better water quality. In River 1A, DO concentrations are consistently high, ranging from 9.05 mg/L in February to 9.80 mg/L in August, which supports healthy aquatic organisms. Conversely, in River 1B, DO values are lower and fluctuate between 8.40 mg/L in May and 10.50 mg/L in August, which still supports life but may indicate a more stressed system, particularly in the summer when oxygen demand is higher.

The biochemical oxygen demand measures the amount of oxygen consumed by microorganisms in breaking down organic material. In River 1A, the BOD values range from 0.48 mg/L in February to 1.04 mg/L in May, indicating low organic pollution. However, in River 1B, BOD levels are notably higher, especially in February, when it reaches 3.12 mg/L. This suggests a higher presence of organic contaminants in the water, which can deplete oxygen levels and disrupt aquatic life if left unchecked.

Another important indicator of the overall pollution load is COD. In River 1A, COD levels fluctuate around 17.43 mg/L in November to 25.01 mg/L in February, which indicates the presence of pollutants that require oxygen to break down. In River 1B, COD values are slightly lower but still concerning, ranging from 6.64 mg/L in November to 12.71 mg/L in August. These elevated values suggest that the river is impacted by pollutants that may not be easily biodegradable. Only the parameter—chemical oxygen demand indicates the presence of oxidizable chemical substances, such as inorganic compounds.

Regarding the microorganisms in the water at the source, fungi (*Mucor* spp.) and yeasts were identified, which indicate organic pollution. Downstream, because acidic mine waters are extremely toxic to most life forms, with low pH and high concentrations of heavy metals, the deposit on the filter inhibited the growth of microorganisms.

For the study, a sampling point of the Roșia Montană River was monitored monthly in the central area of the locality during 2023. Table 4 presents the values of the microbial load and nutrient regime over time.

Exceedances of the permitted limits are observed in the river water samples taken from Roșia Montană area. Exceedances are recorded especially in the summer months. The highest recorded value of TVC was 640 colonies in August 2023, and the highest values of nitrates, nitrites, and ammonium ions were in June and July.

### 2.3. Characterization of the Vegetation in the Roșia Montană Area [31]

In the area of the locality Roșia Montană, habitats of grasslands with a wide variety of plant species and well-conserved habitats have been identified, corresponding to the following Natura 2000 habitats: 6210—Semi-natural dry grasslands and scrubland facies on calcareous substrates (*Festuco-Brometalia*), in complex with rock grasslands, which conserve four rare species from the family *Orchidaceae*; 6410—Molinia meadows on calcareous, peaty or clayey-silt laden soils (*Molinion caeruleae*), with mosaic vegetation of hay meadows with *Sanguisorba officinalis*, Meadows with Molinia on calcareous, peaty, or clayey soils. Other types of grassland habitats, without Natura 2000 correspondence, are represented by birch glades (conserving species such as *Parnassia palustris* and *Gladiolus imbricatus*), hay meadows with *Festuca pratensis* and *Arrhenatherum elatius*, dominated by *Brachypodium pinnatum*, and partially abandoned hay meadows with *Molinia caerulea* and *Sanguisorba officinalis*.

Among the shrubland habitats with Natura 2000 correspondence, we mention: 4030—European dry heaths; 6230*—Species-rich *Nardus* grasslands, on siliceous substrates in mountain areas; 6430—Hydrophilous tall herb fringe communities of plains and of the montane to alpine levels; 6520—Mountain hay meadows, 6510—Lowland hay meadows (*Alopecurus pratensis*, *Sanguisorba officinalis*). Other types of shrubland habitats, without Natura 2000 correspondence, are represented by hazel scrublands (*Corylus avellana*).

Among the forest habitats with Natura 2000 correspondence, we mention: 91V0—Dacian Beech forests (*Symphyto-Fagion*), 9110—*Luzulo-Fagetum* beech forests, 9130—*Asperulo-Fagetum* beech forests, 91E0*—Alluvial forests with *Alnus glutinosa* and *Fraxinus excelsior* (*Alno-Padion*, *Alnion incanae*, *Salicion albae*). Other types of forest habitats, without Natura 2000 correspondence, are represented by: willow scrublands (*Salix triandra*) and Danubian communities with *Typha angustifolia* and *T. latifolia*. Other types of habitats with Natura 2000 correspondence are represented by: 7140—Transition mires and quaking bogs, 8220—Siliceous rocky slopes with chasmophytic vegetation, 8230—Siliceous rock with pioneer vegetation of the *Sedo-Scleranthion* or of the *Sedo albi-Veronicion dillenii*, while habitats without Natura 2000 correspondence include: anthropogenic communities with *Polygonum aviculare*, *Lolium perenne*, *Sclerochloa dura*, and *Plantago major*, anthropogenic communities with *Agropyron repens*, *Arctium lappa*, *Artemisia annua*, and *Ballota nigra*.

The tailings dumps and areas that were exploited in the past, without vegetation or with ruderal vegetation, cover significant areas in this region. The pioneer plant communities, of small surface area, are made up of species such as *Tussilago farfara*, *Crepis setosa*, *Poa bulbosa*, *Poa compressa*, *Galium aparine*, *Medicago lupulina*, and *Erigeron acer*. In some areas, hygrophilous pioneer communities appear, with *Typha angustifolia*, *Phragmites australis*, and *Calamagrostis epigeios*. The flora of the tailings heaps is poor in species, even when compared to other ruderalized areas. Most of the pioneer dicotyledons fail to colonize the unstable surfaces.

Among the anthropized habitats in the area, we mention: Anthropogenic communities with *Polygonum aviculare*, *Lolium perenne*, *Sclerochloa dura*, and *Plantago major*, a habitat characteristic of intensively trampled surfaces, forming strips along roads and paths, with species such as *Polygonum aviculare*, *Lolium perenne*, *Plantago major*, and some more resistant species of mountain hay meadows (*Dactylis glomerata*, *Centaurea indurata*, *Prunella vulgaris*); Anthropogenic communities with *Agropyron repens*, *Arctium lappa*, *Artemisia annua*, and *Ballota nigra*, a habitat characteristic of abandoned agricultural land; Pioneer woody vegetation, mostly on tailings (with *Betula pendula*)—birch scrublands represent the natural pioneer vegetation in the upper nemoral zone. It presents a varied flora, depending on the ecotope, but the birch (*Betula pendula*) predominates in all cases, accompanied in the upper layer (5–10 m) by a few species of shrubs (*Frangula alnus*, *Salix cinerea*, more rarely *Crataegus* sp., *Sambucus racemosa*, *Populus tremula*). Birch stands on old tailings and sterile rocks have an herbaceous layer formed by oligotrophic species and usually acidophilic ones, generally dominated by *Deschampsia flexuosa* or *Agrostis capillaris*, locally by *Luzula sylvatica*, *Poa nemoralis*, and among dicotyledons, *Veronica officinalis*, *Hieracium pilosella*, *H. bauhinii*, *Hieracium murorum*, etc. On smaller areas with deeper soil, *Chamaenerion angustifolium*, *Cirsium rivulare*, *Scirpus sylvaticus*, *Holchus lanatus*, etc., appear; Pine plantations with *Betula pendula* on tailings are made up of black pine (*Pinus nigra*) and Scots pine (*Pinus sylvestris*) mixed with *Betula pendula*. Generally, the herbaceous layer is made up of species like bilberry (*Vaccinium myrtillus*, *Vaccinium vitis-idaea*), heather (*Calluna vulgaris*, especially in the woodland edge area), *Genista sagittalis*, *Avenella flexuosa*, and horst; Lakes surrounded by hygrophilous vegetation—companion species present in several communities: *Phragmites australis*, *Lysimachia vulgaris*, *Solanum dulcamara*, *Stachys palustris*, *Symphytum officinale*. Associated with them, in the form of a narrow band, appear reedbeds—Danubian-Dacian communities with *Carex elata*, *C. rostrata*, *C. riparia*, and *C. acutiformis*.

The Roșia Montană area offers a favorable natural environment for beekeeping due to the diversity of the honey-bearing flora, respectively the purity of the mountain environment. The predominant flora consists of forests, pastures, or meadows that create an ideal habitat for bees. This diversity makes the area attractive to beekeepers and to protect biodiversity. At the same time, traditional practices for cultivating vegetables used in human nutrition are still preserved. Thus, in Table 5 the plant species that grow in the geographical space of Roșia Montană are presented, most of which have honey-bearing potential and human consumption [32].

Roșia Montană is an area of remarkable biodiversity, located in the Apuseni Mountains, Romania, surrounded by forests, meadows, and traditional agricultural lands. The ecosystems in the region include a wide range of plant species, many of which are specific to this mountain environment. Vegetation includes deciduous forests, conifers, alpine meadows, and endemic species, as well as several medicinal plants and vegetables traditionally cultivated in local gardens. This biodiversity is supported by traditional agricultural and pastoral practices, which help maintain natural habitats and protect local species.

The analysis of samples was carried out using X-ray fluorescence spectrometry. To determine the degree of pollution of the vegetation, we chose lead, copper, and cadmium as indicators of pollution. Table 6 presents the values obtained for the samples analyzed in the Roșia Montană area.

The water content of fresh carrots (*Solanum tuberosum*) is between 88–90%, in onions between 85–90%, in potatoes 75–80% and in kohlrabi approximately 90%. In their dehydrated form, in onions, potatoes, and kohlrabi the water content varies between 5–10%, and for carrots between 5–8%.

For the calculation of the conversion factor (CF), the formula CF = (100% − water content in the unprocessed state of vegetable %)/(100% − water content in the processed state of vegetable %) was applied. Considering the average value of 86% of the initial water content of vegetables, respectively 7% for the final one, the CF obtained was 0.15. The heavy metal value for vegetables before dehydration was determined by multiplying the value obtained by the conversion factor.

From Table 6 lead concentrations in samples of mixed vegetables grown in the Roșia Montană area exceeded a limit of 0.2 mg/kg (according to the latest consolidated version from 2023 to Regulation (EC) no. 1881/2006) in samples 1 and 5 to 9, confirming the fact that pollution is a relevant environmental issue. The highest values (0.72 and 0.79 mg/kg) were in the samples taken in NW and the center of the town. Exceedances were also recorded in the case of cadmium in 5 of the 9 samples, located in approximately the same locations as in the case of lead, the maximum value (0.17 mg/kg) being the one recorded in the sample taken from the center of the city. This can be explained by the fact that the maximum allowed concentrations were also exceeded in the soil samples, so there is soil pollution in the sampling area, which is transferred to vegetation. The values highlighted the potential adverse effects on the health of the local population by ingestion of more than one trace element.

Copper was not found in high concentrations in the samples taken. It is an essential micronutrient for all living things. However, too high or too low concentrations of copper in the diet can cause health problems, but it is naturally present in many foods. Regarding doses, the European Food Safety Authority (EFSA) scientific committee concluded that copper retention is not expected to occur with an intake of up to 5 mg per day and established an acceptable daily intake (safe level) of 0.07 mg/kg of body weight for the adult population [36].

### 2.4. Physico-Chemical Analysis of Propolis

The analysis of the physico-chemical composition of propolis is important for determining its quality, especially when it is considered an indicator of the pollution of the area. The results of the physico-chemical characterization of the raw propolis samples analyzed in the Roșia Montană area can be found in Table 7 and Table 8.

Regarding the water activity, the samples recorded values between 0.62 and 0.71. The humidity varied between 5.33% and 7.67%. Regarding the hygroscopicity of the samples, it provides information about their ability to absorb and retain moisture from the environment, which was between 12.9 and 13.8, and the melting point was between 62.7 and 65.2 °C. The melting point is essential to evaluate the authenticity, composition, purity, and quality of propolis. The density of propolis depends on the botanical source and geographical region, with the recorded values falling between 0.982 and 1.159 g/cm^3^. All physical parameters are important for the quality of propolis, including the dry matter parameter which falls between 92.33 and 94.82%.

For the chemical parameters analyzed, no significant differences were found for the propolis samples from the Roșia Montană area. Therefore, the ash content varies between 2.5 (sample 1) and 3.28 g/100 g of product. It reflects the content of minerals and inorganic elements. It is an indicator of mineral purity and composition. For the wax content, different values are recorded depending on the sampling area. The lowest amount was 25.84%, and the highest was 46.33%. High amounts of wax can indicate a lower proportion of biologically active compounds, such as polyphenols and flavonoids. The solubility values of water vary between 10.9 and 12.55%. This parameter determines how easily the active compounds from propolis can be extracted in aqueous solutions, being important for phytotherapy and the food supplement industry. Volatile oils ranged from 0.2 to 0.4%, contributing to the specific aroma of propolis and may have antimicrobial and antioxidant properties. The oxidation index (between 10.9 and 13.1 s) indicates the resistance of propolis to oxidation. Low values indicate increased sensitivity to oxidative degradation, which may affect the stability of active compounds. The insoluble residue in ethanol ranged from 16.2 to 17.1% and represents the amount of material insoluble in ethanol. It is an indicator of the purity of propolis. High content can indicate the presence of impurities or less bioactive components. Phenols and flavonoids are powerful antioxidants that contribute to the antimicrobial, antioxidant, and anti-inflammatory properties of propolis. The lowest phenol content was found in sample S6: 129.6 mg GAE/g, and the highest was found in sample S5: 193.4 mg GAE/g. Flavonoids registered values between 68.59 mg QE/g (S6) and 87.84 mg QE/g (S4). The antioxidant capacity of propolis to inhibit 50% of free radical activity varies between 0.086 and 0.964 µg/mL. The lower the IC_50_ value, the stronger the antioxidant activity of propolis.

### 2.5. Metal Cations in Propolis

Monchanin et al. [37] showed the harmful effects of metal pollutants (at a historic mining site) especially arsenic, on the behavior and cognition of honeybees, even at low levels. The results indicated neurodevelopmental problems and raised serious concerns about the health of bee populations in areas polluted with potentially harmful elements.

Toxic particles adhere to the bodies of bees or are ingested. In addition, chemical analysis of bee products has shown accumulations of metal cations in regions affected by mining activities in Romania, such as the Aries River basin, where concentrations of Cu^2+^, Zn^2+^, and As^3+^ have increased due to mining spills.

The amounts of the trace elements of metal cations obtained (Pb^2+^, Cu^2+^, Cd^2+^, Zn^2+^, As^3+^, Sr^2+^) for all the samples analyzed, as shown in Table 9, are expressed in mg/kg.

The metal content in propolis varies significantly between samples and is not regulated by law. However, the values obtained correlated with those of metal cations in soil, water, and vegetables providing information on the degree of pollution and the geographical origin of propolis samples, reflecting the impact of anthropogenic activities and environmental conditions on their composition. Lead has the highest content in S3 (10.14 mg/kg) and the lowest in S8 (4.19 mg/kg). Copper is the most abundant in S3 (6.32 mg/kg) and the lowest in S8 (1.29 mg/kg). Cadmium reaches a maximum in S9 (1.149 mg/kg) and a minimum in S6 (0.048 mg/kg). Arsenic is highest in S3 (1.037 mg/kg) and the lowest in S7 (0.182 mg/kg). Strontium has extreme values in S3 (12.06 mg/kg) and S8 (4.16 mg/kg). Zinc is maximum in S5 (6.0 mg/kg) and minimum in S1 (3.1 mg/kg). These data reflect the compositional differences of propolis depending on its source from different regions of the Roșia Montană mining area.

## 3. Discussion

Generally, larger quantities of pollutants accumulate in the superficial soil layers (0–20 cm), the data being confirmed by the literature [38]. Lead accumulates in the soil and can affect human health and biodiversity. The high persistence of lead in the soil could be a result of its low mobility and high adsorption properties, which makes it difficult for natural processes to degrade or remove it. High levels of lead have been associated with mining activities in the area. Mining activities can introduce lead into the environment at higher concentrations, significantly altering its natural concentration in soil and potentially leading to long-term contamination. Lead has a high persistence in the soil compared to other pollutants and is low in the parent material: 8–10 ppm in igneous rocks, and 20–26 mg/kg in sedimentary rocks [39]. Representation in uncontaminated soils: 15–110 mg/kg. Contamination is exclusively from anthropogenic sources: mining, ore preparation, non-ferrous metallurgy, sludge and wastewater, chemical, and organic fertilizers, or vehicle exhaust gases [40]. Adsorption at the soil level is mainly influenced by pH and cation exchange capacity (dependent on organic soil colloids). Absorption and translocation in plants vary with species, substrate concentration, soil reaction, and season. Lead is very difficult to remove by elution. At the soil level, lead is found most frequently in the form of sulfate (PbSO_4_), most often bound to the organic phase of the soil, in association with Fe and Mn oxides or carbonates [41]. Certain parameters and concentrations of heavy metals can fluctuate significantly depending on the season. Temperature influences the volatility and solubility of chemicals. Precipitation can influence their transport in the aquatic environment or in the soil. During the rainy season, the increase in the amount of water can favor the leaching of chemicals from the soil into surface waters. At the same time, the biological cycles of plants and organisms in ecosystems can influence the concentrations of heavy metals in plant material or in the soil.

In all 9 analyzed points, the zinc content does not show a high value. Zinc is an essential element in plant nutrition, a component of some enzyme systems, and plays a role in the synthesis of nucleic acids. This is a very mobile and bio-accessible metal for vegetables. Zinc’s mobility in soils and its bioavailability to plants may explain its important role in plant nutrition, although its presence in the environment may remain relatively stable due to natural cycling processes. The total zinc content in soils is influenced by the parent material (residual layer), with an average content at the lithosphere level of 80 mg/kg. Its origin is generally from the atmosphere or using fertilizers and pesticides in agriculture [42].

Its adsorption at the soil level is favored by clay minerals and humic compounds. The availability of zinc for plants is determined by the character and level of exchangeability and/or solubility, imprinted by adsorbed colloids and soil pH. In soil, zinc forms complexes with chlorides, phosphates, nitrates, and sulfates. The formation of these complexes may regulate the amount of free zinc available to plants, as well as its mobility in the soil. It seems that the forms of ZnSO_4_ and ZnHPO_4_ are the most important that contribute significantly to the zinc concentration in soils, and these complexes increase the solubility of zinc. This explains why the application of zinc ammonium sulfate as a fertilizer increases the accessibility of zinc to plants. It forms soluble complexes with fulvic acids, which lead to increased zinc mobility [43].

Soil pollution with mobile copper was not recorded at all the points investigated. The content of mobile copper in suspension in the Roșia Montană area reached a maximum of 23.0 mg/kg in the soil sample taken from the east of the area. In general, lower values of the copper content are found in all sampling points. Copper loading of soil is not a consequence of atmospheric pollution but is due to mining exploitation in the area.

Copper, like other metal cations in high concentrations, is toxic to the environment and acts as an enzyme inhibitor [44]. In general, copper toxicity in agricultural soils is due to human activities, accumulations being the result of soil contamination by excessive treatments with Bordeaux mixture, copper-based fungicides, or because of the activity of the non-ferrous metallurgy industry. Copper is a facultative activator of plant enzyme systems. Its presence is also likely due to contamination from the non-ferrous industry, the use of copper-based fungicides, and the use of sludge in agriculture [45].

Our results are consistent with other studies that have highlighted the presence of metal cation contamination in soil in mining areas [46,47]. Polluting soil with lead, zinc, iron, manganese, and copper represents a widespread environmental challenge closely related to mining activities.

Our results obtained for Pb^2+^ content in propolis from the Roșia Montană area (ranging from 4.19–10.14 mg/kg) are in the range with those reported by Roman et al. [48], who found values between 0.39 and 19.86 mg/kg in an industrialized area of Poland. While the concentration of Cd^2+^ in the samples from Roșia Montană ranges from 0.05 to 0.16 mg/kg, the dispersion of results in Poland was much wider, varying between 0.01 and 0.89 mg/kg. The maximum Cu^2+^ content in Roșia Montană was 6.32 mg/kg, whereas in Poland a value of 16.38 mg/kg was determined, with an average of 7.12 mg/kg. The arsenic content in propolis averaged 0.75 mg/kg, with individual samples ranging from 0.01 to 1.92 mg/kg, while in Roșia Montană the average was 0.63 mg/kg.

In studies conducted over several years, Vakhonina et al. [49] reported an average value for Pb^2+^ of 5.62 mg/kg, which is consistent with the current study, where the average Pb content is 6.56 mg/kg. The average Cd^2+^ content in Romanian samples was 0.23 mg/kg, while the maximum value in the investigated Russian region was 0.19 mg/kg. Arsenic content varies from 0 to 1.04 mg/kg. The strontium content in the Russian propolis samples ranges from 2.1 to 21.01 mg/kg, while in the samples from Roșia Montană, the values range from 4.16 to 12.06 mg/kg.

Pollution leads to the degradation of organic matter in the soil, significantly hindering humification processes, a phenomenon with severe repercussions for the overall ion exchange capacity of the soil [50]. The mineral degradation of highly and excessively polluted soils is reflected in the breakdown and dispersion of soil colloids, particularly in the upper horizons. Over time, this results in the loss of the soil’s ability to bind the absorption complex of aggregates. Structural degradation has also contributed to the emergence and intensification of specific erosion processes in the region. Metal cations retained by the organic and mineral fractions of the soil significantly restrict biological activity, leading to the inhibition of nitrification processes, which are essential for maintaining soil fertility [51].

Acid mine drainage constitutes a significant anthropogenic source of heavy metal discharge on a global scale, representing a primary driver of heavy metal pollution risks in surrounding water bodies [52]. The chemical oxygen demand indicates substances that can be oxidized both under cold and hot conditions, under the action of an oxidant. Organic substances are oxidized under hot conditions and inorganic substances are under cold conditions. The increase in the number of organic substances in water or their appearance at a given time is synonymous with water pollution with germs that usually accompany organic substances. In any case, its presence in water favors the long-term persistence of germs, including pathogenic ones.

Arsenic concentrations are well above the permitted limits downstream of the Roșia Montană River, as it is a common byproduct of gold mining. It is one of the most hazardous metal cations of global environmental concern due to its potential toxicity [53]. Copper and zinc, both metals, are found in high concentrations in the soils and waters surrounding mining operations [47]. Surface and underground workings, waste and development rocks, tailings ponds, as well as water infiltration in these gold mining areas, result in significant volumes of metals such as Zn^2+^, Ni^2+^, Pb^2+^, As^2+^, Cu^2+^, and sulfate ions in river and stream ecosystems [54].

The chemical analyses carried out as part of the study indicate contamination of the river water due to mining activities. The pH values are acidic, and high concentrations of metals: Fe^2+^, Zn^2+^, Cu^2+^, and Mn^2+^ were obtained from the water of the rivers in the area [55].

The sediments from the Aries River—whose tributary is the Abrud River, were highly contaminated with Cd^2+^, Cu^2+^, and As^3+^, considerably with Zn^2+^, moderately with Pb^2+^ and Ni^2+^, and low with Cr^2+^ [56]. Their impact on the water quality of the entire watershed in the area [57].

Ammonia appears as a result of water pollution with organic substances, which undergo decomposition, being the first term of the degradation of nitrogenous substances. Its presence indicates recent pollution. Nitrites appear as a result of pollution with organic substances, either by partial oxidation of the amine radical or by the reduction of nitrates. Their presence indicates older water pollution, but together with high concentrations of ammonia shows that the pollution is continuous [58]. The main source of environmental pollution in the Roşia Montană area is acidic water. The exposure of sulfur-containing rocks to the action of oxygen and water leads to the formation of a weak solution of sulfuric acid that dissolves metal cations in the rock and, together with them, eventually reaches the surface or groundwater without any treatment, thus leading to water pollution. By draining these waters into the flowing waters of the area, their acidity increases, and, in sufficiently high concentrations, they can destroy aquatic fauna and flora [59].

The mining activities in the Roșia Montană area have led to severe pollution of the Roşia and Corna streams and, in addition, the Abrudel stream with acidic waters, the color of the waters, including those in these tributaries, being visible. The surface waters affected by previous mining, including the Abrudel stream, are so polluted that they are useless to communities and the existence of fish or other aquatic life is excluded [60].

From the study carried out on the assessment of the impact of mining and mine waters in the Roșia Montană area on its biocenosis, it is found that the water in the area has a strongly acidic character and is polluted with heavy metals at high concentrations. Water with a high load of metal cations flows through historic mining galleries, reaching directly into watercourses without being treated or purified beforehand. Thus, the water source from the Roșia stream has a significant contribution to pollution with Fe^2+^, Cu^2+^, Cd^2+^, Zn^2+^, and As^3+^, the results obtained being in accordance with research in the literature [61,62].

The neurological impacts of lead and arsenic are well-documented in scientific literature [63,64]. Excessive levels of copper and zinc in the human body have been associated with adverse gastrointestinal effects, including vomiting, diarrhea, stomach cramps, and nausea. In addition, copper has been implicated in liver damage and kidney dysfunction. Elevated zinc levels can negatively affect pancreatic function, alter protein metabolism, and contribute to the development of arteriosclerosis [65].

Many studies examine the translocation of heavy metals from various sources into the environment. These metals accumulate in soil sediments and are subsequently absorbed by plants or managed through adaptation and remediation by specific plant species [66]. Factors such as soil acidity and nutrient availability influence the transfer of heavy metals from soil to plants. By addressing these factors, targeted measures can be implemented to mitigate the impact of heavy metal contamination on industrial food products cultivated in affected regions and beyond [67].

These findings highlight a significant gap in environmental monitoring, which could lead to undetected contamination and potential risks to public health and ecosystems. The use of soils or water from the Roșia Montană area without knowledge of the contamination level can lead to the accumulation of metals in the food chain. As a result, plants growing in this area may be consumed by the population and animals, leading to the accumulation of metals in their bodies. In the Roșia Montană area, there are no environmental policies in place to monitor heavy metal concentrations in soil, water, or plants. Additionally, there are no available databases or maps identifying contaminated areas.

Using MATLAB software, experimental data were processed and analyzed, obtaining a statistical mathematical model that describes the variation of the water nutrient regime as a function of the global microbial load, over time, for the studied samples.

**Figure 1:**
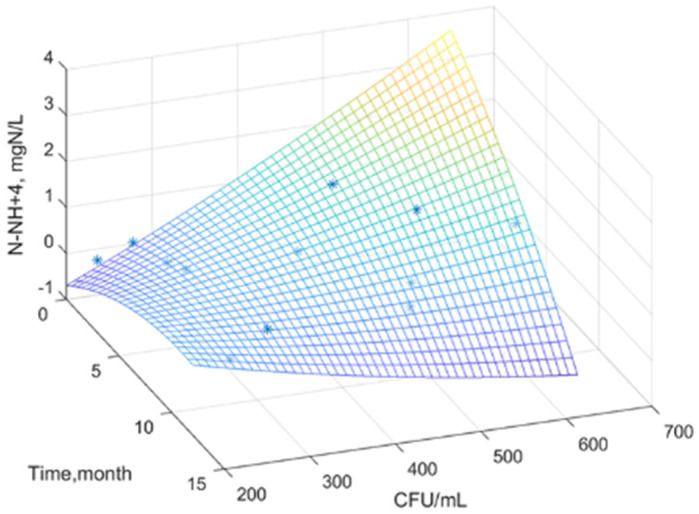
Variation of the ammonia nitrogen content depending on the microbiological load of water over time.

**Figure 2:**
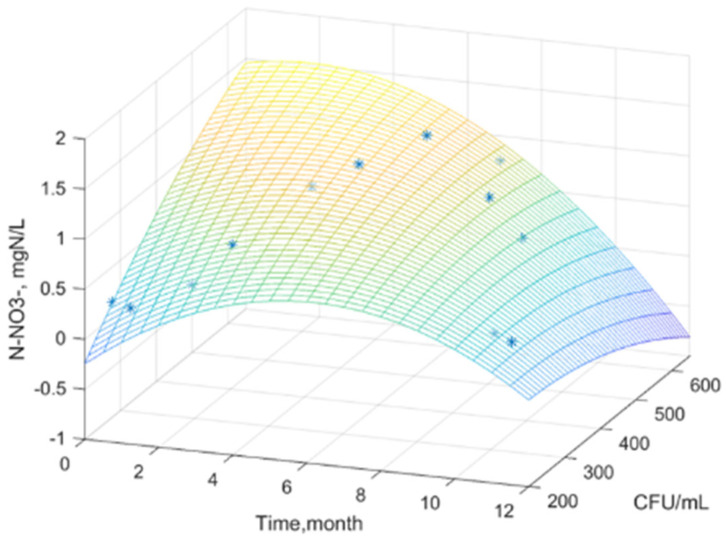
Variation of nitrate ion concentration depending on the microbial load of the water over time.

**Figure 3:**
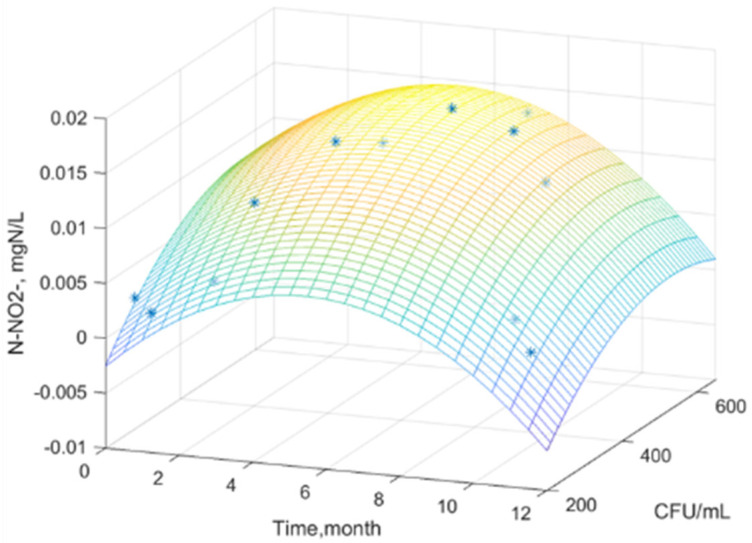
Variation of nitrite ion concentration depending on the microbial load of the water over time.

Figure 1, Figure 2 and Figure 3 show the surfaces that correlate the three variables where: water nutrient regime, x—time, and y—total viable count. Real experimental data were used to build the surface model. The graphical representation was used to determine the mathematical model that highlights the relationships between the experimental variables and to highlight the general trends of the variation of the dissolved inorganic nitrogen concentration.

The generated surface represents an interpolation, a mathematical model of the data. The color gradient indicates variations in the concentration of dissolved inorganic nitrogen as a function of the density of the microbial load over time. It is observed that the concentration of dissolved inorganic nitrogen in all three figures tends to reach a maximum at an intermediate time (approximately 6 months) and a moderate value of TVC/mL (approximately 200–300). At extreme values of time and TVC/mL, the concentration of dissolved inorganic nitrogen is low.

The equations of the statistical models obtained (Table 10) can effectively serve as tools for predictive analysis [68].

The equations of the statistical models obtained can serve as reliable predictive tools. They enable the estimation of the nutrient regime in water based on the time of year when the sample is collected and the microbial load.

Dispersion σ^2^, standard deviation σ, correlation coefficient R, and accuracy coefficient R^2^ were used as indicators of model adequacy (Table 11).

A good correlation between the experimental values and the proposed equation is observed, indicated by the very good coefficient correlation. The correlation parameters support the strong predictive capacity of these statistical models, confirming their practical applicability.

A more elaborate statistical analysis that would establish a direct correlation between the heavy metal content in soil, plant roots, propolis collected by bees, and river water is not possible because each of these elements is influenced by different sources and factors. Soil and water can be contaminated by distinct sources, such as industrial pollution, agricultural activities, or atmospheric deposition, and heavy metals are not evenly distributed in the environment. Plants have varying capacities to absorb heavy metals depending on their species, soil pH, and the type of metal, while propolis is a complex product collected by bees from various plant sources, not just from the cultivated plants in the area. Moreover, heavy metals in river water may originate from sources unrelated to the local soil, and seasonal and climatic variations further influence their concentrations, making a direct correlation even more difficult.

Monchanin et al. [69] demonstrate that bees are unable to detect low concentrations of metals (arsenic, lead, or zinc) found in flowers, thus pollution with these is a major threat to pollinators.

Vegetation—as a pollution indicator—is a useful tool in assessing the negative impact of pollutants on the environment, having the ability to reflect changes in air, soil, and water quality in areas affected by industrial activities, such as mining [70].

The effects of pollutants in mining areas on vegetation can help monitor a wide range of environmental factors and provide valuable information on ecosystem health. Plants are sensitive to chemical changes in the environment, and the accumulation of metal cations, such as arsenic, cadmium, or mercury, in soil and water can affect the photosynthesis process, growth, and development of vegetation [71]. In addition, atmospheric pollution with fine particles or toxic gases can lead to leaf discoloration, reduced resistance to diseases and pests, and even plant death. Therefore, the analysis of the state of vegetation in these areas can provide a rapid and efficient index to identify and assess the impact of industrial pollution on the environment.

Table 12 presents the *p*-values obtained from Tukey’s test to assess the significance of differences in heavy metal content (Pb^2+^, Cu^2+^, Cd^2+^) across different environments (propolis, soil, water, and vegetables).

There is a significant difference between the Pb^2+^ content in propolis and that in soil (*p* = 0.001). The observed differences are not random, indicating a connection between the amount of Pb^2+^ in the soil and that in propolis in the investigated area. However, there are no significant differences between the Pb^2+^ concentration in propolis and water (*p* = 0.899) or between propolis and vegetables (*p* = 0.899).

For Cu^2+^ and Cd^2+^, there is a significant difference between its content in propolis and that in water (*p* = 0.001), highlighting a clear variation in Cu^2+^ and Cd^2+^ content between these environments. However, regarding the Cu^2+^ and Cd^2+^ content between propolis and soil (*p* = 0.899 and *p* = 0.658) or between propolis and vegetables (*p* = 0.899), no significant differences were observed.

A satisfactory correlation is found between the presence of metal cations in propolis and their content in the soil, mixed vegetables, and water in the area, considering the fact that the flight radius of a bee is approximately 3 km around the hive.

Water activity and humidity of the propolis are the parameters that allow the determination of preservation, microbial propagation (indicates whether it is a proper environment for the majority of microorganisms), and the occurrence of chemical reactions of the product [72]. The humidity of the samples is an indicator of the quality and stability of propolis.

Flavonoids exhibit a wide range of biochemical properties; however, their most widely characterized attribute in nearly all flavonoid groups is their ability to function as antioxidants. This antioxidant activity is largely determined by the specific arrangement of functional groups within their core nuclear structure [73]. The structural characteristics of flavonoids, determined by molecular modeling, influence their activity in vegetables, including due to the presence of pollutants. Because of their specific chemical structure, flavonoids easily chelate metal ions and create complex compounds [74].

The variations in heavy metal content in propolis are closely linked to the proximity of pollution sources. For instance, lead, zinc, copper, and arsenic often accumulate in the soil near mining operations due to the release of pollutants during extraction and processing. In these areas, the metal content in plants, including the resins collected by bees for propolis production, is likely to be elevated due to increased soil contamination and higher bioavailability of metals. The potential pathways of heavy metal accumulation in propolis are influenced by the interaction between soil, water, and plants. Heavy metals can enter the soil through natural processes like mineral weathering or anthropogenic activities such as mining, agriculture, or industrial emissions. Once in the soil, metals like lead, zinc, copper, and arsenic can be absorbed by plants, particularly through their roots, depending on factors like soil pH, organic matter content, and metal solubility. These metals can then enter the plant tissues, including the resins and other plant exudates that bees collect. Additionally, water bodies in the surrounding environment can act as conduits for heavy metals, with rainfall and runoff potentially leaching metals from contaminated soils into nearby rivers and streams. This water can then indirectly contribute to heavy metal contamination in plants through irrigation or the absorption of metals by aquatic plants, which may later be incorporated into propolis. The accumulation of these metals in propolis reflects the complex interactions between soil contamination, plant uptake, and environmental factors, ultimately linking the ecosystem’s physical and biological components to the accumulation of these harmful substances.

The limitations of the study refer to the small number of samples collected, which may affect the generalizability of the results. Additionally, the effects of temporal variability across different times of the year can influence heavy metal concentrations, as climatic and biological conditions change annually. This may lead to fluctuations in contamination levels, and the study results could be influenced by these seasonal differences.

## 4. Materials and Methods

### 4.1. Study Area

The Arieș River basin, located in the northern part of the Metaliferi Mountains, has been subjected to extensive mining activities over the past 2000 years. Precious and non-ferrous metals were extracted both through underground mining, as seen in Roșia Montană, and open-pit mining, such as in Roșia Poieni and Roșia Montană. Although mining operations in these areas have largely ceased or decreased in recent years, the potential environmental contamination risk persists. This is due to the substantial amounts of tailings stored in settling ponds and waste heaps located near riverbanks, which represent ongoing sources of pollution of surface and groundwater, soil, and air in the area [75].

The commune of Roșia Montană, which has an area of 4200 hectares, is located in northwestern Romania. It is in a mountainous area of the Apuseni Mountains. The predominant relief is rugged, characteristic of mountain regions, with altitudes varying between high hills and lower mountain peaks. This mountainous area is rich in mineral resources, including gold and other metals, which have been exploited over the centuries [76].

Since 2021, the village of Roșia Montană has been on the UNESCO World Heritage List [77] and has also been listed among the List of World Heritage in Danger [78]. As a result of these measures, any mining activity in Roșia Montană is prohibited.

**Figure 4:**
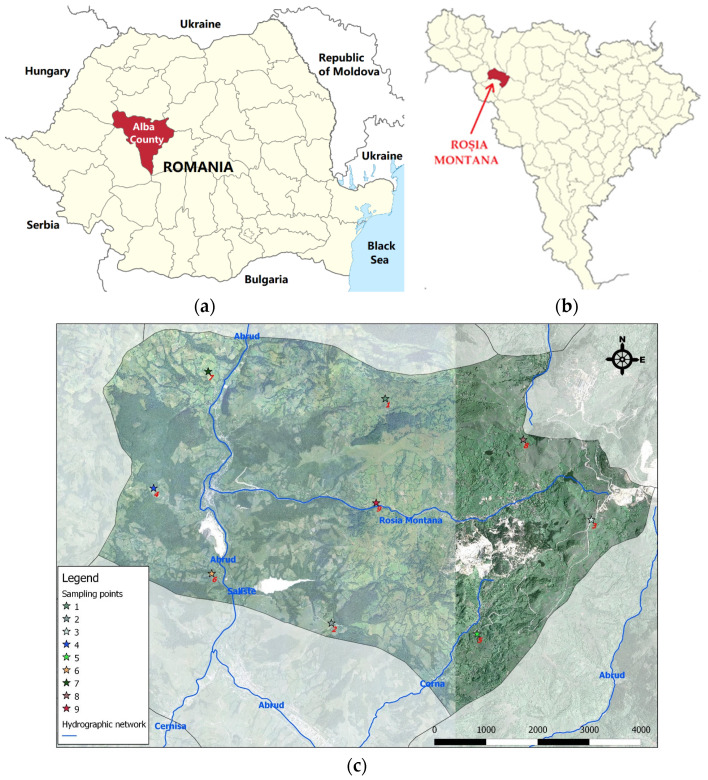
The map of Romania and Alba County—(**a**). The location of the study area—Roșia Montană commune—(**b**). The sampling points—(**c**).

Figure 4 shows the map of Romania and Alba County, respectively, the study area—Roșia Montană commune, located in the north-west of the county.

The map in Figure 4c was created using the open-source software QGIS (Version 3.1.0), utilizing vector and raster data obtained from the website https://geo-spatial.org, as well as vectorized data by the author. The points were added based on GPS points collected in the field at the time of sample collection, using the Android mobile application Spyglass.

### 4.2. Sample Collection and Preparation

Sampling was carried out to understand the dynamics of the chemical parameters in the soil and water, and physico-chemical ones from propolis, specifically to track seasonal variability, and bioavailability of heavy metals.

For the microbiological analysis (total number of microorganisms and identification of fungi), the collection of samples was done monthly, respectively quarterly in order to follow their evolution depending on the ambient temperature, as it is known that in summer months the development of microorganisms is very intense, compared to those in winter.

#### 4.2.1. Soil Sampling and Preparation

Soil sample collection was carried out from pre-selected random points, considering the cardinal directions and the presence of an access road to facilitate faster sample collection. An effort was made to cover the entire area, potentially affected by mining activities that have taken place in the region for over 2000 years, as in some areas, traces of these activities are no longer visible. Soil samples were taken from cultivated land to assess the risk of heavy metal bioaccumulation in vegetables and propolis, which can serve as an indicator of pollution in mining areas.

Soil samples were taken from 9 areas (center, all cardinal points, and between them) within the Roșia Montană commune, with four sampling points (elementary samples located at distances between 5–20 m) established in each area. The soil was collected in accordance with the Romanian standard 7184/1–84 [79]. The sampling was carried out with stainless-steel sampling probes from a depth of 15 cm, this being the depth range at which the roots of the vegetables under study were located. The soil was stored in PVC bags and transported to the laboratory.

The 4 elementary samples extracted from each area were mixed, obtaining an average sample from which the laboratory sample was extracted using the quarter method.

The preparation of laboratory samples for analysis consisted of removing plant debris, crushing, drying at room temperature, and sieving using a sieve with a mesh size of 2 mm. The samples were initially air-dried and subsequently subjected to thermal treatment in an electric oven at 40 °C for approximately 30 min. The resulting fine powder was then stored at room temperature for further analysis. All subsequent tests were performed in triplicate by the same operator and under the same laboratory conditions, the results being expressed as the average of the three tests.

#### 4.2.2. Water Sample Collection and Preparation

Samples were collected from two points in the river (at the source—1A and downstream of the locality—1B) below the surface of the water, using 500 mL of sterile glass vials. From each point, one sample was collected. For the vials used for the determination of metal cations, 2 mL of concentrated HNO_3_ was added to preserve the metals and to avoid precipitation. All subsequent tests were performed in triplicate, the results being expressed as the average of the three tests.

#### 4.2.3. Sampling and Preparation of Vegetables

The vegetables (the part above ground—root/bulb/tuber) subjected to analysis consist of carrot (*Daucus carota*), kohlrabi (*Brassica oleracea* var. *gongylodes*), onion (*Allium cepa*), potato (*Solanum tuberosum*) harvested from the area of the 9 soil sampling points, thus obtaining 9 plant mix samples. The vegetables were chosen for the analysis of heavy metals in the Roșia Montană area due to their ability to accumulate heavy metals in the edible parts, as they are commonly grown by locals and have a root system that directly interacts with potentially contaminated soil. The preparation of samples for analysis was like that for soil, the difference consisting only in the stage of sieving the samples through a sieve, using one with a mesh diameter of less than 1 mm. For the analysis, well-homogenized mixes of plant material were used with the same amounts of each vegetable. All subsequent tests were performed in triplicate, the results being expressed as the average of the three tests.

#### 4.2.4. Propolis Sampling and Preparation

The collection was carried out by beekeepers from the surfaces of the hive, scraping the frames and inner walls with a scraper. A sample constitutes the quantity taken from 10 hives. About 15–25 g of propolis were taken from each hive. These were mixed to form the analysis sample. The sampling was carried out in September 2024. Propolis samples were taken from apiaries located approximately 500 m from the location of soil and vegetable sampling. After collecting, foreign bodies were removed. Subsequently, it was stored in a dark glass container and kept cool and dark. The propolis samples produced by the Romanian bee (*Apis mellifera carpatica*) were taken from apiaries located in the 9 sampling points for soil and plant material. From each point, one sample was collected. All subsequent tests were performed in triplicate, the results being expressed as the average of the three tests.

### 4.3. Chemicals and Reagents

Nitric acid (HNO_3_ 65%), Perchloric acid (HClO_4_ 70%), Barium Chloride (BaCl_2_ · 2H_2_O, for analysis, ≥98%), Potassium iodide (KI 99.995 Suprapur), Sodium thiosulfate (Na_2_S_2_O_3_ solution, c(Na_2_S_2_O_3_) = 0.01 mol/l (0.01 N) Titripur), Sulfuric acid (H_2_SO_4_ 98%), Potassium permanganate (c(KmnO_4_) = 0.02 mol/l (0.1 N) Titripur), Ethanol (96%), Oxalic acid dihydrate Suprapur ((COOH)_2_ · 2 H_2_O), Potassium permanganate (KMnO_4_), Potassium acetate (CH_3_COO-K, purity 99%), Sodium carbonate (Na_2_CO_3_) were obtained from Merck, Germania. Manganese (II) sulfate monohydrate (MnSO_4_ · H_2_O, ACS reagent, ≥98%, DPPH (2,2-diphenyl-1-picrylhydrazyl, purity 95%) were obtained from Sigma-Aldrich (St. Louis, MO, USA).

### 4.4. Physico-Chemical and Microbiological Analysis of Samples

#### 4.4.1. Soil Sample Analysis

The soil samples were subjected to laboratory analysis to determine the concentration of metals. The concentration of Pb^2+^, Cu^2+^, Cd^2+^, Zn^2+^, and As^3+^ in the soil samples was determined in the laboratory by X-ray fluorescence spectrometry (XRF), using an ARL Quant’X EDXRF spectrometer (ThermoFisher Scientific, Waltham, MA, USA). The XRF method validation was conducted in accordance with relevant guidelines: ISO 16934 [80], ASTM standards, and quality assurance guidelines for analytical laboratories.

To prepare the samples for analysis, from the oven-dried samples at a temperature of 105 ° C for 48 h, then ground and sieved through a 1 mm sieve, a quantity of 5 g of soil was mortared and pelletized using a laboratory press model Pellet Press PP 25. The concentration of metals in the soil samples was calculated using UniQuant software (version 5.0).

#### 4.4.2. River Water Analysis

To evaluate the water quality of the Roșia Montană River and its pollution level, a series of parameters were determined, namely: pH, metal content (Cu^2+^, Cd^2+^, Zn^2+^, As^3+^, and Fe^2+^), sulfate content, dissolved oxygen, biochemical oxygen consumption, chemical oxygen consumption, nutrient content (ammonium, nitrites, and nitrates), as well as microbiological load. Validation of non-standardized methods has already been done in the laboratory through repeatability, reproducibility, and measurement of uncertainties.

*pH values*: The pH of the water samples was measured using a HANNA HI-9812-5 portable pH meter (Hanna Instruments GmbH, Graz, Austria).

*Heavy metals*: Water samples were filtered through medium porosity filter paper, previously washed with nitric acid (d = 1.4) diluted 1+1, to adjust the pH to ≤2. The determination of metals was carried out on the agitated and filtered sample. The standard solutions were prepared from reference materials. These solutions were used to construct the calibration curve and periodically verify reference samples against the calibration curve. 200 mL of water samples were digested with 5 mL of di-acid mixture HNO_3_:HClO_4_: 9:4 ratio on a hot plate and filtered using Whatman No. 42 filter paper and made up the volume to 50 mL by double distilled water for the analysis of five metal cations using an atomic absorption spectrophotometer (AA700, Perkin Elmer, Waltham, MA, USA). For the determination of metal cation content (Pb^2+^, Cu^2+^, Cd^2+^, Zn^2+^, As^3+^, and Fe^2+^), water samples were analyzed by flame atomic absorption spectrometry, using an air-acetylene mixture as an oxidant. The parameters for determining the metal content by atomic absorption spectrophotometry [81,82,83,84,85] were for: (1) Pb^2+^ (mg/kg)—wavelength: 283.3 nm, hollow-cathode lamp (Lead), width of slit: 0.5 nm, intensity of the lamp: 5 mA; (2) Cu^2+^ (mg/kg)—wavelength: 324.7 nm, hollow-cathode lamp (copper), width of slit: 0.5 nm, intensity of the lamp: 4 mA; (3) Cd^2+^ (mg/kg)—wavelength: 228.8 nm, hollow-cathode lamp (cadmium), width of slit: 0.5 nm, intensity of the lamp: 4 mA; (4) Zn^2+^ (mg/kg)—wavelength: 213.9 nm, hollow-cathode lamp (zinc), width of slit: 1 nm, intensity of the lamp: 5 mA; (5) As^3+^ (mg/kg)—wavelength: 193.7 nm, hollow-cathode lamp (arsenium), width of slit: 0.5 nm, intensity of the lamp: 10 mA; (6) Fe^2+^ (mg/kg)—wavelength: 372 nm, hollow-cathode lamp (iron), width of slit: 0.1 nm, intensity of the lamp: 5 mA; (7) Sr^2+^ (mg/kg)—wavelength: 460.7 nm, hollow-cathode lamp (strontium), width of slit: 0.5 nm, intensity of the lamp: 10 mA.

The metal content was expressed in milligrams per liter and was calculated using the following formula: Metal = *c* × *r* [mg/L], where: *c* = metal content in the measured solution (mg/L); *r* = dilution factor (100 mL/sample volume analyzed).

*Sulfates*: The determination of sulfates was performed using the turbidimetric method, based on the precipitation of sulfate ions in the form of barium sulfate using barium chloride (BaCl_2_ crystals) as a precipitation reagent. The light transmitted by the turbid solution was measured using a spectrophotometer at a wavelength of 420 nm, using cuvettes with an optical path of 10 mm. The sulfate concentration was determined on the basis of the calibration curve drawn previously, the result being expressed in mg/L SO_4_^2+^ [86].

*Nutrient regime*: The biochemical oxygen demand represents the amount of oxygen consumed by microorganisms over a period for the biochemical decomposition of the organic substances contained in water. The established standard time was 5 days at a temperature of 20 °C.

The Winkler analysis method was used to determine the *dissolved oxygen* (DO) and the *biochemical oxygen demand* (BOD) in the analyzed water. The method consists of the oxidation of Mn(OH)_2_ by the reaction between MnSO_4_ (50%) and HO^−^ ions added to the water sample, to manganese hydroxide, which in an acidic medium removes iodine from potassium iodide (KI) in an amount equivalent to that of oxygen dissolved in water, followed by titration with 0.1N Na_2_S_2_O_3_.

The determination of BOD consists of determining the oxygen consumed for 5 days by microorganisms in water by the difference between the amount of dissolved oxygen found in the water sample at the time of collection and after 5 days of collection. The difference between the dissolved oxygen concentration of the sample that was not subjected to incubation and the average of the dissolved oxygen concentrations kept under incubation for 5 days represents the BOD of the analyzed sample [87,88].

To determine the *chemical oxygen demand* (COD) in the analyzed water, the potassium permanganate method was used. Potassium permanganate oxidizes organic substances in water in an acidic and hot environment, and the remaining excess permanganate is determined with oxalic acid. Thus, 100 mL of water to be analyzed, 5 mL of sulfuric acid 1:3, and 10 mL of 0,01N KMnO_4_ were added to an Erlenmeyer flask. The mixture was boiled for 10 min, then the excess permanganate was removed with 10 mL of 0,01N oxalic acid, and the resulting solution was titrated with potassium permanganate until a persistent pale pink color appeared [89].

*The determination of dissolved inorganic nitrogen*: Rapid spectrophotometric determination was carried out using the Spectroquant NOVA 60 (Merck KGsA, Darmstadt, Germany) spectrophotometer (SQ) in conjunction with SQ-specific kits, which include reagents and reaction tubes [90].

*Ammonium*: Kit SQ domain 0.010–2 mg/l NH_4_-N or 0.01–2.58 mg/l. A volume of 0.5 mL of the sample was added to the reaction tube and thoroughly homogenized. A dose of NH_4_-1K was added to the reaction tube, which was then sealed, shaken, and allowed to rest for 15 min before the measurement was recorded. In highly alkaline solutions, ammonia nitrogen exists predominantly as ammonia, which reacts with hypochlorite ions to form monochloramine. The monochloramine subsequently reacts with a substituted phenol, producing a blue indophenolic derivative.

*Nitrates*: Kit SQ domain 1.0–50.0 mg/dm^3^ NO_3_-N or 2.2–79.7 mg/dm^3^ NO_3_^−^. A volume of 0.5 mL of the test sample was transferred into the tube. A volume of 1 mL of NO_3_^−^1K was added, and the mixture was shaken for one minute and then allowed to rest for 10 min. In a sulfuric acid solution, nitrate ions react with a benzoic acid derivative to produce a red nitro compound.

*Nitrites*: Kit SQ 0.02–1.00 mg/dm^3^ NO_2_-N or 0.07–3.28 NO_2_- NO_2_ 10 mm tube. A volume of 5 mL of the sample was added to the tube, followed by a micro palette knife of NO_2_-1 reagent, which was shaken until completely dissolved, with a reaction time of 10 min. In an acidic solution, nitrite ions react with sulfanilic acid to form a diazonium compound, which subsequently reacts with *N*-(1-naphthyl) ethylenediamine dihydrochloride to produce a violet-red nitro compound.

*Microbiological analysis*: Determination of the total number of bacteria growing at 37 °C (mesophyll) [91,92]: the method involves inoculating a 2 mL sample into 10–15 cm^3^ of nutrient agar culture medium containing yeast extract (Bio Trend, Koln, Germany), melted and cooled to 45 °C. The inoculation was performed in a Petri dish, and after the medium had solidified, the plates were incubated at 37 ± 2 °C for 44 ± 4 h. The total viable count (TVC) expressed in colony-forming units (CFU)/mL was determined by direct counting.

*Identified Microorganisms Genus*: the species identification was performed using a Hund Wetzlar H600LL microscope (Helmut Hund GmbH, Wetzlar, Germany), connected to a PC and operated with Pinnacle TV Center software (https://pinnacle-tvcenter-pro.software.informer.com/).

#### 4.4.3. Analysis of Plant Material

The analysis of the plant material consisted of determining the concentration of Pb, Cu, and Cd by atomic absorption spectrometry using an AA700 spectrometer (Perkin Elmer, Waltham, MA, USA).

For the analysis by AAS, 0.5 g of sample was mineralized in concentrated HNO_3_, using a microwave digester—model TRANSFORM 680 microwave digestion system, manufactured by Aurora Instruments (Vancouver, BC, Canada).

After digestion, the samples were allowed to cool, then filtered using Whatman No. 42 filter paper (Whatman International Ltd., Maidstone, UK), and diluted to a volume of 50 mL with deionized water [93].

#### 4.4.4. Propolis Analysis

- Qualitative physico-chemical parameters

*Water activity* (a_w_) values were determined at 25 °C using an Aquaspector AQS-2-TC water activity meter (Nagy Messsysteme GmbH, Gäufelden, Germany). Each sample was measured in triplicate [94,95].

*The moisture content* (water content) was assessed using an A&D ML50 Moisture Analyzer (A&D Technology, Inc., Japan).

*Hygroscopicity*: the method for determining hygroscopicity proposed by Cai and Corke was used [96]. Ten g sample of propolis was mixed with 100 mL of ultrapure water and centrifuged at 70 rpm using a Centra CL2 centrifuge (Thermo Fisher Scientific Inc., Waltham, MA, USA). The obtained extract was filtered using Whatman Filter Paper Grade No. 1 (Whatman International Ltd., Maidstone, UK) and stored at −50 °C. The frozen propolis was subsequently dried using a condenser-type freeze dryer (TOPT-12A vacuum freeze dryer, Xi’an, China). Two g of propolis powder was stored at 25 °C in a container with a saturated Na_2_SO_4_ solution (81% RH) for 7 days, after which it was weighed. Hygroscopicity was quantified as the amount of water absorbed per 100 g of propolis, expressed in grams.

*Melting point* (MP): a 3–4 mm layer of propolis was settled into a capillary tube with a 1 mm diameter and sealed at one end. A thermometer, embedded in paraffin, was placed adjacent to the propolis layer, with paraffin serving as the embedding medium for the thermometer. The temperature of the paraffin bath gradually increased, and the temperature at which the propolis in the capillary tube completely melted was recorded as the melting point [97].

*Density*: the homogenized propolis sample was weighed, and its volume was determined by measuring the amount of water it displaced in a 50 mL graduated cylinder. The density of the sample was subsequently calculated by dividing the weight by the volume. This process was repeated for three individual samples.

*Dry matter* analysis was performed at 105 °C using an A&D ML50 Moisture Analyzer (A&D Technology, Inc., Ibaraki, Japan) [98].

*The ash content* (total mineral substances) was determined by ashing 10 g of raw propolis in a crucible at approximately 525 °C [98].

*Wax content*: 5 g of dry propolis were sequentially extracted with petroleum ether at 40–60 °C for 3 h using a Soxhlet extractor. The samples were then oven-dried at 100 °C for 3 h and allowed to cool until they reached a constant weight [98].

*Water solubility*: a 2 g sample of propolis was centrifuged at 5000 rpm for 5 min in a Centra CL2 centrifuge (Thermo Fisher Scientific Inc., Waltham, MA, USA) with 20 mL of distilled water. A 5 mL aliquot was then dried at 105 °C in an oven. Solubility was determined by weighing the sample before and after the drying process [99].

*Volatile oils* (VO): a distillation flask was charged with 250 mL of distilled water and 50 g of crushed raw propolis. The volatile oils, carried by the water vapor, were collected in the graduated collector tube, forming the upper layer. Distillation continued until the volume of the supernatant remained constant [100].

For the *oxidation index* (OI), 2.5 mL of ethanol was mixed with 0.1 g of raw propolis. After one hour, 50 mL of water was added, and the mixture was filtered. To 2 mL of the resulting filtrate, a 20% sulfuric acid solution and a drop of 0.1 N potassium permanganate were added. Using a stopwatch, the time in seconds was recorded for the solution to become colorless, with the timer starting while the mixture was continuously shaken. The insoluble residue collected on the cellulose thimble at the end of the Soxhlet extraction was then dried in an oven at 80 °C until a constant weight was achieved [101].

*Ethanol-insoluble residue* (EIR): 1 g of propolis and 10 mL of ethanol (96%) were stirred for 24 h, filtered on Whatman paper no. 41 in a vacuum; the solid residue (ethanol insoluble material) was dried in a glass desiccator to constant weight [102].

Total phenolic content (TPC)—Folin-Ciocalteu method: fifty grams of dried raw propolis were dissolved and homogenized in 150 mL ethanol 96% using a Climo-Shaker ISF1-XC shaker incubator (Kuhner, Basel, Switzerland) at 600 rpm, at 40 °C for 24 h (kept away from light), and the mixture was then filtered on filter paper. For the Folin-Ciocalteu assay, 1 mL of the ethanolic extract was mixed with 5 mL of distilled water and an equivalent volume of Folin-Ciocalteu reagent (1:1 diluted with water). After incubation for 5 min, 4 mL of 7.5% Na_2_CO_3_ solution was added, and the mixture was incubated in the dark for 30 min at room temperature in the dark to allow color development (blue complex formation). The absorbance of the samples was measured at 765 nm using a Lambda 20 UV-VIS Spectrophotometer (Perkin Elmer UV/VIS, Washington, DC, USA), with distilled water as the blank. The total phenolic concentrations were determined by comparison to a standard gallic acid calibration curve of known concentrations (0–200 µg/mL). The results were expressed as mg of gallic acid equivalent (GAE) per gram of propolis extract (mg GAE/g) [103,104].

*Total flavonoid content* (TFC)—Aluminum chloride method: to 1 g of propolis, 2.5 mL of 96% ethanol was added, and the mixture was centrifuged for 24 h at 200 rpm using a Centra CL2 centrifuge (Thermo Fisher Scientific Inc., Waltham, MA, USA). After centrifugation, 25 mL of 80% ethanol was added to the supernatant. Then, 0.5 mL of the ethanolic extract was mixed with the following reagents: 0.1 mL of 10% AlCl_3_, 0.1 mL of 1M CH_3_COO-K, 1.5 mL of 95% ethanol, and 2.5 mL of distilled water. The mixture was then kept in the dark for 50 min (to allow the formation of the flavonoid–AlCl_3_ complex). Absorbance was measured at 425 nm using a Lambda 20 Perkin Elmer UV/VIS spectrophotometer (Waltham, MA, USA). The total flavonoid content (TFC, expressed as mg of quercetin equivalents (QE)/100 g of propolis) was determined using a calibration curve prepared with a standard quercetin solution (0–100 µg/mL) [105].

*Antioxidant activity* (AA): DPPH was utilized as a free radical to assess the radical scavenging activity (RSA) of propolis. An alcoholic extract of propolis was prepared at room temperature by homogenizing propolis with a 70% ethanol solution (1:100 *w*/*v*) for 24 h. After complete evaporation of ethanol, the resulting concentrated dry substance was used for further analysis. Two solutions were prepared: one containing 0.6 mg/mL of propolis and the other containing a 0.1 mM DPPH ethanolic solution. The absorbance was measured at λ = 515 nm using a Lambda 20 UV-VIS Spectrophotometer (Perkin Elmer UV/VIS, Washington, DC, USA). Absorbance (A) was recorded at the start of the reaction and again after 10 and 20 min. Antioxidant activity was then calculated using the following formula: %RSA = (A_DPPH_ − A_sample_)/A_DPPH_ × 100 [106,107]. The free radical scavenging capacity of the propolis samples was expressed as the IC_50_ value. To determine the IC_50_, the radical scavenging activity (RSA) was measured for each propolis sample at five different concentrations: 0.6, 1, 2, 3, and 4 mg/mL.

*Heavy metals*: The determination of the metal content in propolis (Pb^2+^, Cu^2+^, Cd^2+^, Zn^2+^, As^3+^, and Sr^2+^) was performed by flame atomic absorption spectrometry, using a Perkin Elmer AA700 atomic absorption spectrometer. The working parameters of the apparatus are those presented in the description of the method for the analysis of metals in water. For AAS analysis, samples were first mineralized in concentrated HNO_3_ at 80–100 °C for 60 min, until complete dissolution and then filtered. Mineralization was performed using a TRANSFORM 680 microwave digestion system model, manufactured by Aurora Instruments. After mineralization, the samples were aspirated into the flame for the analysis of the metal content. The wavelengths at which the metal content was determined are those presented in the water analysis [108].

### 4.5. Statistical Analysis

Polynomials provide a more comprehensive framework for representing functions and variable relationships compared to linear equations, allowing for more intricate modeling, adaptable curve fitting, and improved predictive accuracy. A non-linear multiple correlation of second degree among 3 parameters was chosen. Based on the experimental determinations, a series of data resulted that can be used to evaluate the comparative effect of the measured parameters by performing a multiple correlation analysis of the content of the nutrient diet, as a dependent variable, based on other parameters, such as microbial load, over time, the last two being independent variables. Therefore, particular attention was given to establishing the relationship among these variables. To develop an accurate predictive model for predicting nutrient (ammonium (NH_4_^+^), nitrates (NO_3_^−^), nitrites (NO_2_^−^)) variation in water, a polynomial multivariable regression analysis was used. The regression parameters were estimated by the method of the least squares that minimize the sum of the squares of the differences between observed and predicted values.

The software used was MATLAB R2018a Software Package (The MathWorks, Inc., Natick, MA, USA). The model accuracy was estimated graphically and by calculating the following evaluation parameters: standard deviation (σ), dispersion (σ^2^), correlation coefficient (R), and determination coefficient (R^2^).

Using analysis of variance followed by a Student’s *t*-test in Origin 2025 (OriginLab Corporation, Northampton, MA, USA), the significance of the values obtained for soil in relation to the alert threshold—sensitive, as well as the metal content in propolis compared to the limits specified in Codex Stan 193/1995, General Standard for Contaminants, was evaluated, with a significance level: *p*-value < 0.01.

Origin 2025 (OriginLab Corporation, Northampton, MA, USA) was used to perform the Tukey test, which was applied to evaluate whether there is a significant relationship between the heavy metal content in propolis and that in water, soil, and vegetables in the Roșia Montană area. The interpretation of *p*-values (*p* < 0.01) helps determine whether the differences between these environments are statistically significant or if the metal levels are comparable.

## 5. Conclusions

Due to the potential negative ecological effects of mining areas, contamination of soil, water, and plants with heavy metals has become a critical environmental concern. The results obtained showed that these are also found in propolis.

The maximum levels allowed in the Roșia Montană area of most trace metals for the soil, water, and vegetables studied exceeded the values of the health-based guidelines. This method provides a detailed picture of the ecological impact of industrial and mining activities. The main cause is the tailings dumps, spills, and sediments from mining tailings ponds in the area. These data contribute to the understanding and management of environmental risks in such areas.

The use of bees as bioindicators has the advantage of wide geographical coverage, collecting samples from various points of the ecosystem. At the same time, they can be a sensitive detection tool for minor chemical changes in the environment, and the methodology would be non-invasive if the sampling and analysis of bee products are relatively simple and do not involve habitat destruction. Bees are a vector and an effective tool for monitoring pollution in areas exposed to mining activities. By monitoring the content of metal cations (such as lead, cadmium, or arsenic) in propolis, levels of contamination with toxic substances can be assessed, since propolis is partly composed of resins collected by bees from plants or surfaces exposed to pollution.

This study highlights that bee products, particularly propolis, serve as valuable bioindicators of environmental pollution. The composition of propolis reflects the accumulation of metal cations present in the surrounding environment, providing information on the extent and type of contamination. This finding underscores the potential of propolis to act as a natural tool to monitor ecological health and detect anthropogenic impacts in mining regions.

## Figures and Tables

**Table 1:** Metal ion concentration values (mg/kg) in soil samples from the Roșia Montană area.

Sample	Sample Place	Pb^2+^	Cu^2+^	Cd^2+^	Zn^2+^	As^3+^
1.	Soil 1—North	91.60 ± 7.32	15.66 ± 2.94 *	3.74 ± 2.04 *	17.41 ± 1.56 *	83.60 ± 9.61 *
2.	Soil 2—South	21.33 ± 2.18 *	8.40 ± 1.22 *	3.10 ± 1.20 *	15.80 ± 2.71 *	77.25 ± 8.83 *
3.	Soil 3—East	56.04 ± 6.21	23.06 ± 5.40 *	2.84 ± 1.25 *	5.16 ± 2.34 *	64.15 ± 6.50 *
4.	Soil 4—West	68.75 ± 5.04	8.19 ± 1.63 *	3.34 ± 5.26 *	12.62 ± 2.49 *	60.32 ± 7.11 *
5.	Soil 5—S-E	34.16 ± 4.91	8.01 ± 1.52 *	2.61 ± 1.97 *	2.53 ± 1.18 *	52.50 ± 5.72 *
6.	Soil 6—S-W	21.90 ± 3.75 *	14.61 ± 2.71 *	3.09 ± 1.74 *	30.00 ± 2.22 *	81.72 ± 7.40 *
7.	Soil 7—N-W	106.43 ± 5.96 *	12.53 ± 2.67 *	3.55 ± 2.53 *	36.16 ± 4.07 *	102.17 ± 17.0 *
8.	Soil 8—N-E	108.32 ± 4.74 *	3.67 ± 1.40 *	3.47 ± 1.05 *	6.12 ± 1.43 *	95.08 ± 12.48 *
9.	Soil 9—Centre	26.30 ± 3.40 *	14.68 ± 2.08 *	3.56 ± 3.68 *	27.59 ± 3.05 *	80.46 ± 10.95 *
Normal Values [20]		20	20	1	100	5
Alert threshold [28]	Sensitive	50	100	3	300	15
	Less sensitive	250	250	5	700	25
Intervention threshold [28]	Sensitive	100	200	5	600	25
	Less sensitive	1000	500	10	1500	50

* The obtained values for soil were reported to the alert threshold—sensitive (the first signal that soil pollution may have a negative impact on agriculture), with a significance level defined as *p*-value < 0.01.

**Table 2:** Results of analyses for monitoring chemical parameters—metalloids (µg/L) of Roșia Montană River water.

Sampling Sites	Month of Sampling	Pb^2+^	Cu^2+^	Cd^2+^	Zn^2+^	As^3+^	Fe^2+^
River 1A—at the source	February	ND	ND	27.08 ± 4.05	48.42 ± 10.03	ND	150.44 ± 40.08
	May	ND	ND	20.33 ± 0.90	65.18 ± 13.19	ND	104.03 ± 71.62
	August	ND	ND	55.72 ± 0.61	51.04 ± 17.11	ND	230.20 ± 98.34
	November	ND	ND	41.16 ± 0.82	53.22 ± 9.31	ND	167.05 ± 43.06
River 1B—in the middle	February	ND	374.08 ± 11.10	50.89 ± 0.40	107.05 ± 25.00	117.20 ± 1.30	23,790.42 ± 308.26
	May	11.00 ± 0.50	820.23 ± 27.09	20.51 ± 0.22	1632.40 ± 62.83	134.44 ± 2.74	38,901.97 ± 5251.04
	August	11.00 ± 0.40	903.47 ± 41.68	60.13 ± 0.53	1890.27 ± 33.62	148.07 ± 1.95	44,024.08 ± 911.63
	November	ND	210.30 ± 18.22	40.09 ± 0.30	1903.08 ± 50.20	75.06 ± 4.22	29,162.36 ± 523.05
Quality class [29]	I	5	20	0.5	100	10	300
	II	10	30	1	200	20	500
	III	25	50	2	500	50	1000
	IV	50	100	5	1000	100	2000
	V	>50	>100	>5	>1000	>100	>2000

Abbreviation: ND—not detectable.

**Table 3:** Values of the chemical and microbiological parameters recorded in the Roșia Montană River at the source and downstream of the locality.

Sampling Sites	Month of Sampling	pH	SO_4_^2−^, mg/L	DO, mgO_2_/L	BOD, mgO_2_/L	COD, mg O_2_/L	Identified Microorganisms (Genus)
River 1A—at the source	February	7.80 ± 0.08	48.22 ± 4.11	9.05 ± 0.29	0.48 ± 0.02	25.01 ± 5.14	It has developed fungi: yeasts and molds (*Mucor* spp.)
	May	7.42 ± 0.07	21.01 ± 2.00	9.47 ± 0.62	1.04 ± 0.31	17.90 ± 3.02	
	August	6.34 ± 0.08	33.16 ± 2.53	9.80 ± 0.33	0.19 ± 0.01	21.22 ± 3.81	
	Novembre	7.05 ± 0.08	29.58 ± 1.82	9.53 ± 0.49	0.57 ± 0.03	17.43 ± 2.66	
River 1B—in the middle	February	3.87 ± 0.05	1307.10 ± 60.34	9.66 ± 0.66	3.12 ± 1.00	10.10 ± 1.90	The deposit in the filter inhibited the growth of microorganisms.
	May	3.59 ± 0.06	1239.47 ± 44.71	8.40 ± 0.41	2.03 ± 0.52	8.20 ± 1.57	
	August	2.80 ± 0.04	1197.88 ± 35.40	10.50 ± 0.58	2.62 ± 0.61	12.71 ± 2.23	
	Novembre	3.76 ± 0.05	1076.29 ± 20.22	9.32 ± 0.97	2.67 ± 0.43	6.64 ± 1.28	
Quality classes [29]	I	6.5–8.5	60	8	3	5	-
	II		120	7	5	10	
	III		250	5	7	20	
	IV		300	4	20	50	
	V		>300	<4	>20	>50	

Abbreviations: SO_4_^2^—sulfate; DO—dissolved oxygen; BOD—biochemical oxygen demand; COD—chemical oxygen demand.

**Table 4:** Experimental results obtained for the water sampled from the Roșia Montană River.

Time [Month]		CFU/mL	NH_4_^+^-N, µg N/L	NO_3_^−^-N, µg N/L	NO_2_^−^-N, µg N/L
January		278	21.40 ± 1.09	144.05 ± 23.20	1.92 ± 0.34
February		224	26.01 ± 1.22	280.43 ± 19.16	2.10 ± 0.42
March		294	35.32 ± 3.16	352.29 ± 36.08	3.74 ± 0.90
April		303	101.18 ± 2.44	761.77 ± 20.35	11.03 ± 0.51
May		422	424.29 ± 37.35	1040 ± 105.00	14.21 ± 0.74
June		450	2050.00 ± 20.91	1222.16 ± 77.14	13.80 ± 0.93
July		536	1507.17 ± 18.05	1303.52 ± 149.04	15.32 ± 1.10
August		640	1162.43 ± 15.40	781.67 ± 40.36	12.93 ± 0.22
September		505	490.20 ± 46.28	850 ± 51.27	14.60 ± 1.07
October		492	248.15 ± 44.37	513.63 ± 24.09	10.53 ± 0. 83
Novembre		312	303.46 ± 58.06	130.55 ± 11.21	2.44 ± 0. 09
Decembre		256	149.33 ± 5.44	242.58 ± 48.07	1.01 ± 0.05
Waters [30]	Salmonid	-	≤40	-	≤10
	Cyprinid	-	≤200	-	≤30
Quality classes [29]	I	-	400	1000	10
	II	-	800	3000	30
	III	-	1200	5600	60
	IV	-	3200	11,200	300
	V	-	>3200	>11,200	>300

Abbreviations: CFU—colony-forming unit; NH_4_^+^-N—ammonium nitrogen; NO_3_^−^-N—nitrate nitrogen; NO_2_^−^-N—nitrite nitrogen.

**Table 5:** Native, cultivated, and melliferous plant species from the Roșia Montană area.

**Deciduous and coniferous trees**	**Deciduous Trees:** *Betula pendula* (Silver Birch), *Frangula alnus* (Alder Buckthorn), *Salix cinerea* (Grey Willow), *Sambucus racemosa* (Red Elderberry), *Populus tremula* (Aspen), *Corylus avellana* (Hazel), *Crataegus monogyna* (Common Hawthorn), *Tilia cordata* (Small-leaved Lime), *Sorbus aucuparia* (Rowan or Mountain Ash), *Prunus spinosa* (Blackthorn), *Rubus* sp. (Brambles), *Rosa* sp. (Rose), *Alnus glutinosa* (Black Alder), *Fraxinus excelsior* (European Ash), *Crataegus* sp. (Hawthorn), *Salix alba* (White Willow), *Salix caprea* (Goat Willow), *Lonicera xylosteum* (Fly Honeysuckle), *Corylus avellana* (Hazel), *Betula pendula* (Silver Birch), *Populus* sp. (Poplar), *Crataegus monogyna* (Common Hawthorn), *Sorbus aucuparia* (Rowan or Mountain Ash) **Coniferous Trees:** *Pinus sylvestris* (Scots Pine), *Pinus nigra* (Black Pine), *Abies alba* (European Silver Fir), *Picea abies* (Norway Spruce).
**Shrubbery** **species**	*Prunella vulgaris* (Selfheal, Heal-all), *Rubus* sp. (Brambles, Blackberries, Raspberries), *Prunus spinosa* (Blackthorn, Sloe), *Sambucus racemosa* (Red Elderberry), *Rosa* sp. (Rose, Wild Rose), *Crataegus monogyna* (Hawthorn, Mayflower), *Lonicera xylosteum* (Tartarian Honeysuckle), *Genista sagittalis* (Broom, Arrow Broom), *Lysimachia vulgaris* (Yellow Loosestrife, Garden Loosestrife), *Lonicera xylosteum* (Tartarian Honeysuckle), *Sorbus aucuparia* (Rowan, Mountain Ash), *Stachys sylvatica* (Wood Betony).
**Herbaceous plants and wildflowers**	*Erigeron acer* (Bitter Fleabane), *Centaurea indurata* (Cornflower, Knapweed), *Ballota nigra* (Black Horehound), *Hieracium pilosella* (Mouse-ear Hawkweed), *Hieracium bauhinii* (Bauhinii Hawkweed), *Hieracium murorum* (Wall Hawkweed), *Chamaenerion angustifolium* (Rosebay Willowherb, Fireweed), *Cirsium rivulare* (River Thistle), *Serratula tinctoria* (Dyer’s Cudweed), *Succisa pratensis* (Devil’s-bit Scabious), *Parnassia palustris* (Grass-of-Parnassus), *Vaccinium myrtillus* (Bilberry), *Vaccinium vitis-idaea* (Lingonberry), *Lysimachia vulgaris* (Yellow Loosestrife, Garden Loosestrife), *Solanum dulcamara* (Bittersweet, Woody Nightshade), *Stachys palustris* (Marsh Woundwort), *Cirsium arvense* (Creeping Thistle), *Cirsium erisithales* (Plumeless Thistle), *Reynoutria japonica* (Japanese Knotweed), *Impatiens glandulifera* (Himalayan Balsam), *Telekia speciosa* (Showy Yellow Daisy), *Geranium palustre* (Marsh Crane’s-bill), *Chaerophyllum hirsutum* (Hairy Chervil), *Lythrum salicaria* (Purple Loosestrife), *Caltha laeta* (Large-flowered Marsh-marigold), *Menyanthes trifoliata* (Bogbean), *Comarum palustre* (Marsh Cinquefoil), *Antennaria dioica* (Common Pussytoes), *Symphytum cordatum* (Heart-leaved Comfrey), *Lamium galeobdolon* (Yellow Archangel), *Galium odoratum* (Sweet Woodruff), *Cardamine glanduligera* (Glandular Bittercress), *Daphne mezereum* (February Daphne), *Dentaria bulbifera* (Toothwort), *Stellaria holostea* (Greater Stitchwort), *Lathyrus niger* (Black Pea), *Lathyrus vernus* (Spring Vetchling), *Scorzonera rosea* (Pink Goat’s-beard), *Polygala vulgaris* (Common Milkwort), *Molinia caerulea* (Purple Moor-grass), *Serratula tinctoria* (Dyer’s Cudweed), *Succisa pratensis* (Devil’s-bit Scabious), *Astrantia major* (Masterwort), *Dianthus superbus* (Large Pink), *Gentiana pneumonante* (Spring Gentian), *Hypochoeris maculata* (Spotted Cat’s-ear), *Alchemilla vulgaris* (Lady’s Mantle), *Trollius europaeus* (Globeflower), *Polygonum bistorta* (Bistort), *Ferulago campestris* (Field Ferulago), *Trifolium pratense* (Red Clover), *Anacamptis pyramidalis* (Pyramidal Orchid), *Gymnadenia conopsea* (Fragrant Orchid), *Campanula patula* (Spreading Bellflower), *Achillea millefolium* (Yarrow), *Trifolium repens* (White Clover), *Leucanthemum vulgare* (Oxeye Daisy), *Mentha longifolia* (Long-leaved Mint), *Geranium phaeum* (Dusky Crane’s-bill), *Colchicum autumnale* (Autumn Crocus), *Crocus banaticus* (Banat Crocus), *Sanguisorba officinalis* (Great Burnet), *Geranium pratense* (Meadow Crane’s-bill), *Centaurea phrygia* (Phrygian Knapweed), *Sedum album* (White Stonecrop), *Sedum acre* (Biting Stonecrop), *Thymus comosus* (Hairy Thyme), *Silene nutans ssp. dubia* (Nodding Catchfly), *Acinos arvensis* (Field Basil).
**Medicinal plants species**	*Tussilago farfara* (Colt’s foot), *Galium aparine* (Cleavers), *Medicago lupulina* (Black medic), *Erigeron acer* (Fleabane), *Prunella vulgaris* (Self-heal), *Arctium lappa* (Burdock), *Ballota nigra* (Black horehound), *Sambucus racemosa* (Red elderberry), *Gentiana pneumonante* (Autumn gentian), *Sanguisorba officinalis* (Great burnet), *Parnassia palustris* (Grass-of-Parnassus), *Telekia speciosa* (Telekia), *Filipendula ulmaria* (Meadowsweet), *Symphytum officinale* (Comfrey), *Corylus avellana* (Hazel), *Crataegus monogyna* (Hawthorn), *Eupatorium cannabinum* (Hemp agrimony), *Cirsium arvense* (Creeping thistle), *Angelica sylvestris* (Wild angelica), *Impatiens glandulifera* (Himalayan balsam), *Lonicera xylosteum* (Fly honeysuckle), *Geranium palustre* (Marsh geranium), *Chaerophyllum hirsutum* (Hairy chervil), *Lythrum salicaria* (Purple loosestrife), *Mentha longifolia* (Long-leaved mint), *Astrantia major* (Masterwort), *Daphne mezereum* (Mezereon), *Galium odoratum* (Sweet woodruff), *Cardamine glanduligera* (Glandular bittercress), *Stellaria holostea* (Greater stitchwort), *Lathyrus vernus* (Spring vetchling), *Pulmonaria officinalis* (Lungwort), *Scorzonera rosea* (Violet salsify), *Nardus stricta* (Nardus), *Polygala vulgaris* (Common milkwort), *Serratula tinctoria* (Saw-wort), *Dianthus superbus* (Fringed pink), *Hypochoeris maculata* (Spotted cat’s ear), *Alchemilla vulgaris* (Lady’s mantle), *Achillea millefolium* (Yarrow), *Trifolium repens* (White clover), *Genista tinctoria* (Dyer’s greenweed), *Leucanthemum vulgare* (Ox-eye daisy), *Mycelis muralis* (Wall lettuce), *Mentha longifolia* (Long-leaved mint), *Senecio paludosus* (Marsh ragwort), *Geranium phaeum* (Dusky cranesbill), *Crocus banaticus* (Banat crocus), *Sanguisorba officinalis* (Great burnet), *Geranium pratense* (Meadow geranium), *Centaurea phrygia* (Knapweed), *Sedum album* (White stonecrop), *Sedum acre* (Goldmoss stonecrop), *Thymus comosus* (Wild thyme), *Acinos arvensis* (Field basil).
**Cultivated** **vegetables**	*Phaseolus vulgaris* (Beans), *Lactuca sativa* (Lettuce), *Satureja hortensis* L. (Thyme).

**Table 6:** Heavy metal content: lead, cadmium, and copper content (mg/kg) in mixed vegetables.

Sample	Sample Name	Pb^2+^	Cu^2+^	Cd^2+^
1	Mixed vegetables 1—North	0.30 ± 0.04	1.15 ± 0.09 *	0.02 ± 0.01 *
2	Mixed vegetables 2—South	0.14 ± 0.03 *	0.73 ± 0.24	0.10 ± 0.02
3	Mixed vegetables 3—East	0.13 ± 0.02 *	1.15 ± 0.13 *	0.01 ± 0.00 *
4	Mixed vegetables 4—West	0.25 ± 0.09	1.08 ± 0.06 *	0.01 ± 0.00 *
5	Mixed vegetables 5—S-E	0.56 ± 0.11	1.27 ± 0.31	0.03 ± 0.01 *
6	Mixed vegetables 6—S-W	0.42 ± 0.04	1.32 ± 0.20 *	0.12 ± 0.04 *
7	Mixed vegetables 7—N-W	0.72 ± 0.07 *	1.51 ± 0.05 *	0.15 ± 0.04
8	Mixed vegetables 8—N-E	0.38 ± 0.06	1.48 ± 0.08 *	0.11 ± 0.02 *
9	Mixed vegetables 9—Center	0.79 ± 0.13	0.80 ± 0.17	0.17 ± 0.04
Maximum levels (mg/kg wet weight) * [33]		0.20	-	0.05 *
Maximum permissible limits in vegetables, mg/kg [34,35]		0.30	40.0	0.10

* The obtained values for vegetables were reported according to the limits specified in Codex Stan 193/1995, General Standard for Contaminants, with a significance level defined as *p*-value < 0.01. Note: For peeled potatoes (*Solanum tuberosum*) the maximum level is 0.10 mg/kg wet weight. According to Regulation (EC) no. 1881/2006 setting maximum levels for contaminants established by the Codex Alimentarius Commission [33]. A total of 54 samples were analyzed (n = 6 analyses for each food item).

**Table 7:** Determination of the physical properties of brown propolis samples from Roșia Montană.

Sample No.	a_w_	Moisture, %	Hygroscopicity, g H_2_O/100 g Propolis	MP, °C	Density, g/cm^3^	Dry Matter, %
S1	0.63 ± 0.08	5.33 ± 0.25	13.3 ± 0.3	65.2 ± 0.3	1.104 ± 0.003	94.67 ± 0.20
S2	0.62 ± 0.09	6.24 ± 0.19	13.8 ± 0.7	62.7 ± 0.2	0.983 ± 0.004	93.76 ± 0.23
S3	0.70 ± 0.04	5.18 ± 0.33	13.0 ± 0.5	63.4 ± 0.3	1.097 ± 0.005	94.82 ± 0.24
S4	0.71 ± 0.10	5.95 ± 0.28	13.5 ± 0.4	64.1 ± 0.2	1.055 ± 0.008	94.05 ± 0.28
S5	0.66 ± 0.07	7.67 ± 0.11	13.7 ± 0.8	63.6 ± 0.1	0.996 ± 0.007	92.33 ± 0.22
S6	0.65 ± 0.03	6.93 ± 0.24	12.9 ± 0.6	64.3 ± 0.2	1.159 ± 0.003	93.06 ± 0.19
S7	0.64 ± 0.05	5.70 ± 0.12	13.1 ± 0.5	65.0 ± 0.3	0.982 ± 0.004	94.30 ± 0.21
S8	0.66 ± 0.06	6.83 ± 0.26	13.4 ± 0.8	62.9 ± 0.2	1.080 ± 0.005	93.17 ± 0.25
S9	0.67 ± 0.09	6.12 ± 0.30	13.7 ± 0.4	63.5 ± 0.2	1.034 ± 0.006	93.88 ± 0.28

Abbreviations: a**_w_**—water activity; MP—melting point.

**Table 8:** Determination of the chemical properties of brown propolis samples from Roșia Montană.

Sample No.	Ash, g/100g	Wax, %	Water Solubility, %	VO, %	OI, s	Ethanol-Insoluble Residue, %	Phenols, mg GAE/g	Flavonoids, mg QE/g	IC_50_, µg/mL
S1	2.50 ± 0.00	25.84 ± 0.57	11.46 ± 0.79	0.4 ± 0.06	12.5 ± 1.4	16.4 ± 0.8	189.4 ± 5.82	84.31 ± 0.09	0.333 ± 0.002
S2	2.85 ± 0.08	37.18 ± 0.81	10.90 ± 0.62	0.3 ± 0.04	12.3 ± 2.1	17.1 ± 0.6	180.8 ± 4.54	78.26 ± 0.07	0.514 ± 0.016
S3	2.96 ± 0.04	40.56 ± 1.06	12.55 ± 0.13	0.2 ± 0.03	11.8 ± 1.1	16.8 ± 0.9	172.9 ± 3.25	78.55 ± 0.08	0.725 ± 0.003
S4	3.15 ± 0.06	33.22 ± 0.38	11.16 ± 0.45	0.3 ± 0.08	12.5 ± 1.9	16.5 ± 0.2	189.5 ± 4.83	87.84 ± 0.11	0.669 ± 0.010
S5	3.28 ± 0.09	46.33 ± 1.05	10.27 ± 0.50	0.2 ± 0.01	10.9 ± 2.0	16.9 ± 0.4	193.4 ± 7.22	86.06 ± 0.08	0.884 ± 0.028
S6	2.55 ± 0.05	37.41 ± 0.58	10.82 ± 0.31	0.3 ± 0.02	11.7 ± 1.3	16.7 ± 0.7	129.6 ± 3.58	68.59 ± 0.09	0.964 ± 0.031
S7	2.73 ± 0.03	31.19 ± 0.71	11.38 ± 0.24	0.4 ± 0.07	11.4 ± 1.5	17.0 ± 0.8	184.3 ± 6.04	82.27 ± 0.25	0.086 ± 0.001
S8	2.69 ± 0.04	32.52 ± 0.44	12.04 ± 0.33	0.2 ± 0.03	12.6 ± 1.7	16.6 ± 0.3	152.2 ± 6.80	70.10 ± 0.16	0.517 ± 0.04
S9	3.08 ± 0.03	28.92 ± 0.67	11.61 ± 0.46	0.3 ± 0.05	13.1 ± 1.8	16.2 ± 0.5	157.1 ± 5.57	74.35 ± 0.36	0.615 ± 0.05

Abbreviations: VO—volatile oils; OI—oxidation index; GAE—gallic acid equivalents; QE—quercetin equivalents; IC_50_—half maximal inhibitory concentration (DPPH assay).

**Table 9:** Minerals content (mg/kg) of propolis samples.

	Metal Cation	Pb^2+^	Cu^2+^	Cd^2+^	Zn^2+^	As^3+^	Sr^2+^
Sample							
S1		8.26 ± 0.68	4.03 ± 0.22	0.16 ± 0.04	3.12 ± 0.19	0.97 ± 0.30	7.78 ± 0.86
S2		7.04 ± 0.52	4.44 ± 0.81	0.15 ± 0.08	5.71 ± 0.36	0.79 ± 0.14	9.92 ± 0.54
S3		10.14 ± 0.87	6.32 ± 0.56	0.14 ± 0.04	4.04 ± 0.60	1.04 ± 0.28	12.06 ± 0.92
S4		5.60 ± 0.42	3.21 ± 0.47	0.14 ± 0.08	3.45 ± 0.36	0.64 ± 0.18	5.48 ± 0.42
S5		8.42 ± 0.33	5.07 ± 0.75	0.15 ± 0.04	6.09 ± 0.65	0.42 ± 0.13	7.75 ± 0.75
S6		5.01 ± 0.47	2.10 ± 0.24	0.05 ± 0.09	3.36 ± 0.48	0.77 ± 0.25	7.32 ± 0.64
S7		4.65 ± 0.63	3.68 ± 0.12	0.10 ± 0.06	4.50 ± 0.22	0.18 ± 0.04	8.40 ± 0.87
S8		4.19 ± 0.30	1.29 ± 0.54	0.07 ± 0.05	3.91 ± 0.99	0.51 ± 0.10	4.16 ± 0.25
S9		5.72 ± 0.90	4.01 ± 0.55	1.15 ± 0.18	3.28 ± 0.17	0.32 ± 0.09	5.01 ± 0.35

**Table 10:** Equations of statistical models.

Variable “z”	Equations of Statistical Models
Ammonium (NH_4_^+^)	z = −2.2480 + 0.4409∙x + 0.0072·y − 0.0001·x·y − 0.0104·x^2^ + 2.9010·10^−6^·y^2^
Nitrates (NO_3_^−^)	z = −1.4030 + 0.3636∙x + 0.0064·y − 4.4004·10^−4^·x·y − 0.0222·x^2^ − 3.0979·10^−6^·y^2^
Nitrites (NO_2_^−^)	z = −0.0172 + 0.0031∙x + 8.8940·10^−5^·y − 5.2599·10^−7^·x·y − 2.7660·10^−8^·x^2^ − 7.7968·10^−8^·y^2^

**Table 11:** Statistical adequacy indicators.

Related to the Variable “z”	σ^2^	σ	R^2^	R
Ammonium (NH_4_^+^)	0.166	0.407	0.617	0.785
Nitrates (NO_3_^−^)	0.020	0.142	0.883	0.940
Nitrites (NO_2_^−^)	0.001	0.001	0.927	0.963

**Table 12:** Tukey’s test *p*-values for heavy metal content in propolis, soil, water, and vegetables.

Treatments Pair	Tukey *p*-Value		
	Pb^2+^	Cu^2+^	Cd^2+^
Propolis—soil	0.001	0.899	0.658
Propolis—water	0.899	0.001	0.001
Propolis—vegetables	0.899	0.899	0.899

## Data Availability

Data is contained within the article.
